# A comprehensive survey of bat sarbecoviruses across China in relation to the origins of SARS-CoV and SARS-CoV-2

**DOI:** 10.1093/nsr/nwac213

**Published:** 2022-10-11

**Authors:** Zhiqiang Wu, Yelin Han, Yuyang Wang, Bo Liu, Lamei Zhao, Junpeng Zhang, Haoxiang Su, Wenliang Zhao, Liguo Liu, Shibin Bai, Jie Dong, Lilian Sun, Yafang Zhu, Siyu Zhou, Yiping Song, Hongtao Sui, Jian Yang, Jianwei Wang, Shuyi Zhang, Zhaohui Qian, Qi Jin

**Affiliations:** NHC Key Laboratory of Systems Biology of Pathogens, Institute of Pathogen Biology, Chinese Academy of Medical Sciences and Peking Union Medical College, Beijing 110730, China; Key Laboratory of Respiratory Disease Pathogenomics, Chinese Academy of Medical Sciences and Peking Union Medical College, Beijing 110730, China; NHC Key Laboratory of Systems Biology of Pathogens, Institute of Pathogen Biology, Chinese Academy of Medical Sciences and Peking Union Medical College, Beijing 110730, China; NHC Key Laboratory of Systems Biology of Pathogens, Institute of Pathogen Biology, Chinese Academy of Medical Sciences and Peking Union Medical College, Beijing 110730, China; NHC Key Laboratory of Systems Biology of Pathogens, Institute of Pathogen Biology, Chinese Academy of Medical Sciences and Peking Union Medical College, Beijing 110730, China; NHC Key Laboratory of Systems Biology of Pathogens, Institute of Pathogen Biology, Chinese Academy of Medical Sciences and Peking Union Medical College, Beijing 110730, China; College of Animal Science and Veterinary Medicine, Shenyang Agricultural University, Shenyang 110866, China; NHC Key Laboratory of Systems Biology of Pathogens, Institute of Pathogen Biology, Chinese Academy of Medical Sciences and Peking Union Medical College, Beijing 110730, China; NHC Key Laboratory of Systems Biology of Pathogens, Institute of Pathogen Biology, Chinese Academy of Medical Sciences and Peking Union Medical College, Beijing 110730, China; NHC Key Laboratory of Systems Biology of Pathogens, Institute of Pathogen Biology, Chinese Academy of Medical Sciences and Peking Union Medical College, Beijing 110730, China; College of Animal Science and Veterinary Medicine, Shenyang Agricultural University, Shenyang 110866, China; NHC Key Laboratory of Systems Biology of Pathogens, Institute of Pathogen Biology, Chinese Academy of Medical Sciences and Peking Union Medical College, Beijing 110730, China; NHC Key Laboratory of Systems Biology of Pathogens, Institute of Pathogen Biology, Chinese Academy of Medical Sciences and Peking Union Medical College, Beijing 110730, China; NHC Key Laboratory of Systems Biology of Pathogens, Institute of Pathogen Biology, Chinese Academy of Medical Sciences and Peking Union Medical College, Beijing 110730, China; NHC Key Laboratory of Systems Biology of Pathogens, Institute of Pathogen Biology, Chinese Academy of Medical Sciences and Peking Union Medical College, Beijing 110730, China; NHC Key Laboratory of Systems Biology of Pathogens, Institute of Pathogen Biology, Chinese Academy of Medical Sciences and Peking Union Medical College, Beijing 110730, China; NHC Key Laboratory of Systems Biology of Pathogens, Institute of Pathogen Biology, Chinese Academy of Medical Sciences and Peking Union Medical College, Beijing 110730, China; NHC Key Laboratory of Systems Biology of Pathogens, Institute of Pathogen Biology, Chinese Academy of Medical Sciences and Peking Union Medical College, Beijing 110730, China; NHC Key Laboratory of Systems Biology of Pathogens, Institute of Pathogen Biology, Chinese Academy of Medical Sciences and Peking Union Medical College, Beijing 110730, China; College of Animal Science and Veterinary Medicine, Shenyang Agricultural University, Shenyang 110866, China; NHC Key Laboratory of Systems Biology of Pathogens, Institute of Pathogen Biology, Chinese Academy of Medical Sciences and Peking Union Medical College, Beijing 110730, China; NHC Key Laboratory of Systems Biology of Pathogens, Institute of Pathogen Biology, Chinese Academy of Medical Sciences and Peking Union Medical College, Beijing 110730, China

**Keywords:** SARS-CoV, SARS-CoV-2, bats, China, origin tracing

## Abstract

SARS-CoV and SARS-CoV-2 have been thought to originate from bats. In this study, we screened pharyngeal and anal swabs from 13 064 bats collected between 2016 and 2021 at 703 locations across China for sarbecoviruses, covering almost all known southern hotspots, and found 146 new bat sarbecoviruses. Phylogenetic analyses of all available sarbecoviruses show that there are three different lineages—L1 as SARS-CoV-related CoVs (SARSr-CoVs), L2 as SARS-CoV-2-related CoVs (SC2r-CoVs) and novel L-R (recombinants of L1 and L2)—present in *Rhinolophus pusillus* bats, in the mainland of China. Among the 146 sequences, only four are L-Rs. Importantly, none belong in the L2 lineage, indicating that circulation of SC2r-CoVs in China might be very limited. All remaining 142 sequences belong in the L1 lineage, of which YN2020B-G shares the highest overall sequence identity with SARS-CoV (95.8%). The observation suggests endemic circulations of SARSr-CoVs, but not SC2r-CoVs, in bats in China. Geographic analysis of the collection sites in this study, together with all published reports, indicates that SC2r-CoVs may be mainly present in bats of Southeast Asia, including the southern border of Yunnan province, but absent in all other regions within China. In contrast, SARSr-CoVs appear to have broader geographic distribution, with the highest genetic diversity and sequence identity to human sarbecoviruses along the southwest border of China. Our data provide the rationale for further extensive surveys in broader geographical regions within, and beyond, Southeast Asia in order to find the most recent ancestors of human sarbecoviruses.

## INTRODUCTION

Coronaviruses (CoVs) are a group of enveloped viruses with a positive single-stranded RNA genome within the subfamily *Orthocoronavirinae*. They are phylogenetically classified into four genera: *Alphacoronavirus* (α-CoV), *Betacoronavirus* (β-CoV), *Gammacoronavirus* and *Deltacoronavirus* [1]. Prior to 2019, there were six known human CoVs (hCoVs), including NL63, 229E, OC43, HKU1, SARS-CoV and MERS-CoV. NL63 and 229E belong to α-CoVs, and the remaining four are β-CoVs. While infection by NL63, 229E, OC43 and HKU1 leads to the common cold, SARS-CoV and MERS-CoV can cause severe pneumonia, even death [2]. NL63, 229E, SARS-CoV and MERS-CoV are thought to originate from different bat CoVs [2–6], of which SARS-related CoVs (SARSr-CoVs) were mainly found in horseshoe bats (Rhinolophidae) and a few other bat species [5,7–14].

In late 2019, a new hCoV, named SARS-CoV-2, was reported in Wuhan, China. As of 16 August 2022, it has resulted in >588 million confirmed cases and >6.4 million deaths, posing a great threat to global health and economy (https://covid19.who.int/). Like SARS-CoV, SARS-CoV-2 belongs to *Sarbecovirus*, a subgenus of β-CoVs, and SARS-CoV-2 also uses human angiotensin-converting enzyme 2 (hACE2) as its entry receptor. Several SARS-CoV-2-related CoVs (SC2r-CoVs), including isolates RshSTT182, RshSTT200, RacCS203, Rc-o139, BANAL-20-52 (103 and 236), RaTG13, RmYN02 and RpYN06, have been identified in various *Rhinolophus* species (*Rhinolophus shameli, R. acuminatus, R. marshalli, R. pusillus, R. cornutus, R. affinis* and *R. malayanus*) from Cambodia, Thailand, Laos, Japan and the southern border area of Yunnan separately [15–21], suggesting that SARS-CoV-2 also likely originated from bats. Moreover, a few SC2r-CoVs were also found in Malayan pangolins seized during anti-smuggling operations [22,23]. However, whether these bat and pangolin species could serve as the direct natural reservoirs and intermediate hosts for SARS-CoV-2, respectively, remains elusive because of remaining genome differences [24]. Efforts to discover bat CoVs with higher sequence identity than previously reported are warranted for further confirmation of bats as the direct natural hosts of immediate ancestors of SARS-CoV and SARS-CoV-2.

In this study, we performed CoV screening of samples from >13 000 bats collected prior to and during the Coronavirus Disease 2019 (COVID-19) pandemic, from 14 provinces of China. We discovered a new recombinant lineage of sarbecoviruses and found a divergence trend for sarbecoviruses with an increase of diversity and identity with SARS-CoV in southwest China, but failed to detect SC2r-CoVs that were closely related to SARS-CoV-2 in all of their genome. These data indicate that SC2r-CoV might be very limited in bats in China, and more extensive surveying is needed in the Indochina Peninsula and the southern border area of Yunnan, and any other under-sampled areas.

## RESULTS

### Hotspot delineation and bat sampling

**Figure 1.**
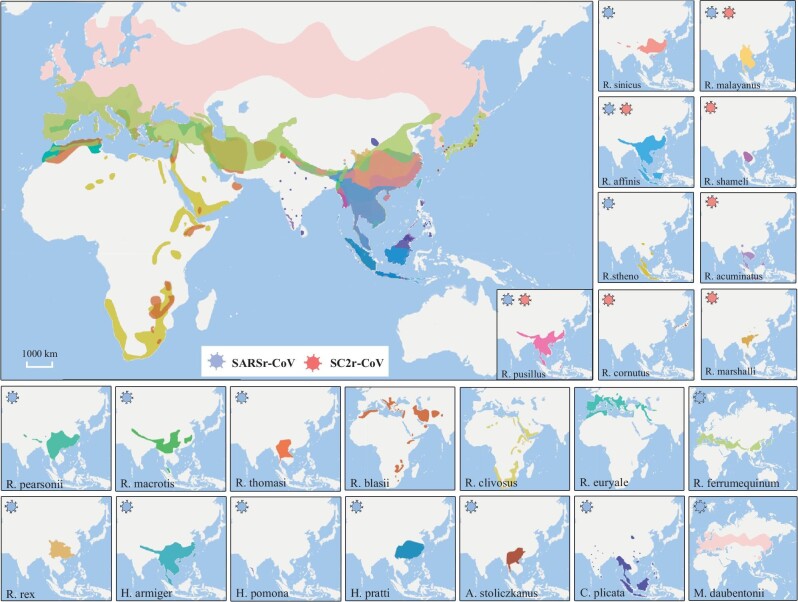
Geographical distributions of bat species carrying sarbecoviruses in Asia, Europe and Africa. The main distribution ranges of *R. sinicus, R. affinis, R. pusillus, R. cornutus, R. malayanus, R. shameli, R. acuminatus, R. marshalli, R. pearsonii, R. macrotis, R. thomasi, R. blasii, R. clivosus, R. euryale, R. ferrumequinum, R. rex, R. stheno, H. armiger, H. pomona, H. pratti, A. stoliczkanus, C. plicata* and *M. daubentoni* are labeled in 23 different colors. The host species once detected for SARSr-CoV and SC2r-CoV are labeled separately. Further information for these species can be found at the International Union for the Conservation of Nature (https://www.iucnredlist.org/). 审图号: GS京(2023)0922号.

To date, a total of >36 000 bat individuals from 100 species across China have been sampled for extensive CoV survey. Sixteen bat species (*Rhinolophus sinicus, R. pusillus, R. ferrumequinum, R. affinis, R. pearsonii, R. macrotis, R. malayanus, R. thomasi, R. rex, R. stheno, Chaerephon plicata, Hipposideros armiger, H. pratti, H. pomona, Aselliscus stoliczkanus* and *Myotis daubentonii)* were reported to carry sarbecoviruses in China (Fig. 1 and Table S1). Among them, *R. rex* is an endemic species of China, *R. ferrumequinum* and *M. daubentonii* are widely distributed in Europa and Asia, while all other species are found in Asia and mainly in South Asia, Southeast Asia (SEA) and southern China. Outside China, ∼7000 bat individuals from 60 species in Asia, and 9500 bat individuals from 108 species in Europe and Africa have been sampled for CoVs as of 2020, out of which 11 species (*Rhinolophus shameli, R. cornutus, R. blasii, R. euryale, R. clivosus, R. acuminatus, R. marshalli, R. ferrumequinum, R. pusillus, R. malayanus* and *Hipposideros galeritus*) were reported to be sarbecovirus-positive (Fig. 1 and Table S1). The host distribution of susceptible species highlights clear differences between where SARSr-CoVs and SC2r-CoVs are found.

**Figure 2.**
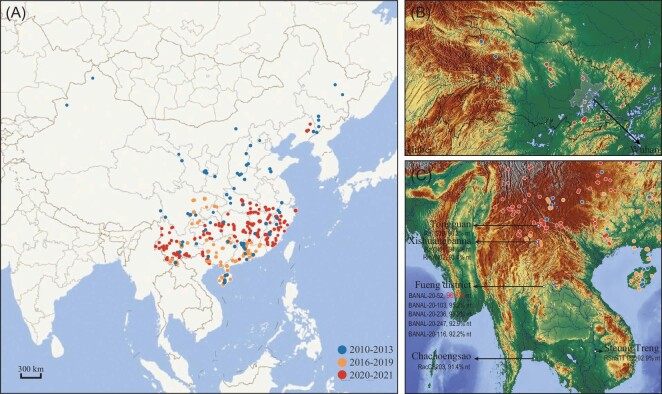
Sites and dates of sample collection. (A) The blue dots represent sampling sites from 2010 to 2013, the orange dots represent sampling sites from 2016 to 2019, and the red dots represent sampling sites from 2020 to 2021. (B) Bat sampling sites on a relief map of mountain areas around Wuhan city. (C) Bat sampling sites on a relief map in or around the Tongguan cave and Xishuangbanna Tropical Botanical Garden. Previously reported SC2r-CoV-related sites are labeled by black arrows. 审图号: GS京(2023)0922号.

Following the survey based on samples of 4440 bats between 2010 and 2013 [25], we extended our study in seven provinces of southern China from 2016 to 2019, with focus on the regions and species hotspots deduced from previous studies (Fig. 2A). After the first report of COVID-19 cases, we continued the project and adapted a much deeper sample collection strategy across the mainland of China. The newly added locations included a series of suspected hotspots in the area around Wuhan city in Hubei province (336 bats of 9 species from 19 sites, 291 of which were *Rhinolophus* bats, Fig. 2B), Zhoushan city in Zhejiang province, Liaoning province and the nine southern provinces and related border regions. It is worth noting that *Rhinolophus* species in and around the abandoned mining cave of Tongguan town, where RaTG13 was found [15], and the karstic caves around the Xishuangbanna Tropical Botanical Garden near the southern border of Yunnan province, close to where RmYN02 and RpYN06 were found [16,21], were also sampled (978 bats of 30 species from 46 sites in Puer city and Xishuangbanna Prefecture; 207 of them were *Rhinolophus* bats, Fig. 2C). In total, our survey covered 703 sampling sites, including 416 old and 287 new sites, where the bat species suspected or confirmed to carry sarbecoviruses are found. Pharyngeal and anal swabs from 13 064 individuals of 54 species and 4 undetermined species were collected (Fig. 2A, Tables S2, S3 and S8). The most commonly sampled species were members of the families Rhinolophidae, Hipposideridae and Vespertilionidae (the habitat/flight distribution maps for these bat species are shown in Fig. 1 and Supplementary Fig. 1).

### CoV screening

All pharyngeal and anal swab samples of bats were combined into 372 pools according to the collection date, sampling point and host species (numbered as P001–P372) (Table S4). Partial RNA-dependent RNA polymerase (RdRp) based PCR results showed that 113 pools were α-CoV-positive, 64 pools were β-CoV-positive and 22 pools were both α-CoV- and β-CoV-positive (Supplementary Fig. 2A). Meanwhile, following next-generation sequencing (NGS), 199 out of 372 pools were found to be CoV-positive (Supplementary Fig. 2C). All CoV-related reads could be classified into five subgenera of α-CoV (*Minunacovirus, Decacovirus, Pedacovirus, Rhinacovirus* and *Nyctacovirus*) and three subgenera of β-CoV (*Nobecovirus, Merbecovirus* and *Sarbecovirus*). Sarbecoviruses were mainly found in *R. sinicus, R. ferrumequinum* and *R. affinis* captured in Guangdong, Yunnan, Fujian, Guangxi, Hubei and Liaoning, with 83.2%–100% nt identities to each other within the 440 nt fragment of RdRp.

To further delineate the prevalence and positive rate of sarbecoviruses, all 1068 individual samples in 44 sarbecovirus-positive pools were selected for sample-by-sample screening, and 146 samples were sarbecovirus-positive (Supplementary Fig. 2B and Table S5). These sarbecoviruses were identified from *R. ferrumequinum, R. sinicus, R. pusillus, R. affinis, R. rex, R. luctus* and *R. siamensis* captured in Guangdong, Yunnan, Fujian, Liaoning, Hunan, Hubei, Jiangxi, Anhui and Guangxi provinces, with 88.8%–100% nt identities to known SARSr-CoVs in the 440 nt fragment of RdRp. Among sarbecovirus-positive samples, 69 out of the 146 samples were selected for genomic sequencing as quasi-species and their whole-genome sequence was used for subsequent analysis.

### Evolution trend derivation for sarbecoviruses

**Figure 3.**
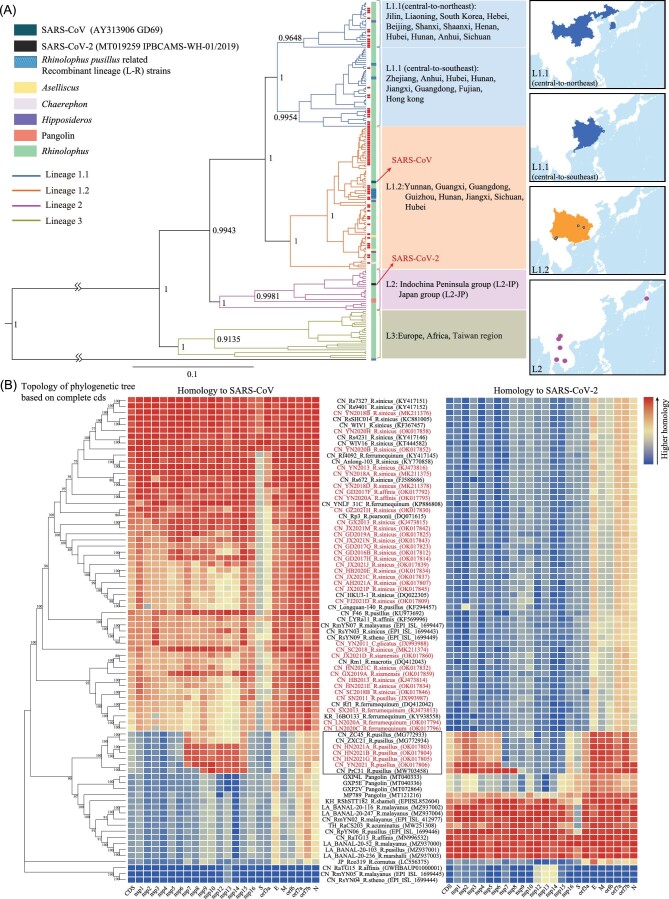
(A) Evolution overview of sarbecoviruses. Maximum-clade-credibility tree inferred from partial RdRp (nsp12) sequences. L1.1 is labeled in blue, L1.2 is labeled in orange, L2 is labeled in violet and L3 is labeled in gray. Node labels represent estimated posterior probability for the main grouping nodes. Distributions of sarbecoviruses in China and surrounding counties are shown by different color lumps identical to the colors of different lineages. All viruses identified in this study are labeled by red boxes. The scale bar behind the tree represents the average number of substitutions per time unit. (B) Homology analysis for non-structure, structure and accessory proteins of sarbecoviruses. Heatmap of the homology analyzes of 78 sarbecoviruses (sequences obtained here are labeled in red font) compared with SARS-CoV (AY313906) and SARS-CoV-2 (MT019529). Columns are scaled by the zero-to-one method, and rows are clustered with the phylogenetic tree based on complete genome sequences. 审图号: GS京(2023)0922号.

By using the newly identified bat sarbecoviruses here, and 123 CoVs available in GenBank or Global Initiative on Sharing All Influenza Data (GISAID) (Table S1), a phylogenetic reconstruction based on the 440 nt RdRp sequences was conducted. The addition of the newly identified CoVs makes the maximum clade credibility (MCC) tree display an obvious clustering trend. Except for one outgroup CoV, identified from *H. galeritus* in Malaysia, all bat sarbecoviruses could be divided into three major lineages: lineage 1 (L1) as SARSr-CoVs, lineage 2 (L2) as SC2r-CoVs and lineage 3 (L3) as sarbecoviruses found in Europe, Africa and Taiwan region (Fig. 3A). The separation between the outgroup CoV and the common ancestor of L1, L2 and L3 likely occurred first, then L3 might have diverged from the ancestral L1–L2 lineage and L1 separated from L2 later. The evolutionary origin of all known sarbecovirus lineages can be traced to Rhinolophid hosts. All sarbecoviruses identified here were clustered into L1. L1 can be further divided into two sublineages, L1.1 and L1.2. L1.1 was mainly found in three bat rhinolophus species (*R. sinicus, R. ferrumequinum and R. pusillus*) and occasionally can also be found in other bat species, including *R. macrotis, R. thomasi, M. daubentonii and Hipposideros armiger*, with different geographical distribution including China and South Korea. Of note, L1.1 can be further separated into two groups, central-to-northeast and central-to-southeast. L1.2 was mainly presented in four bat species (*R. sinicus, R. ferrumequinum, R. pusillus and R. affinis*) and rarely found in other species (*R. pearsonii, R. rex, R. siamensis, R. luctus, R. stheno, A. stoliczkanus, H. armiger, H. pratti* and *H. pomona*) in southern China. L2 was found in *R. pusillus, R. malayanus, R. shameli, R. acuminatus, R. marshalli, R. affinis, R. stheno* and *R. cornutus*, which were scattered across the Indochina Peninsula, the southern border region of Yunnan province, and Japan. L3 contained CoVs found in the *Rhinolophus* species (*R. ferrumequinum, R. blasii, R. euryale, R. hipposideros, R. pusillus and R. clivosus*) from regions outside the mainland of China, including Taiwan of China, Italy, Bulgaria, France, Russia, Luxembourg, Slovenia, Croatia, Rwanda, Uganda and Kenya.

The nucleotide identities of all non-structural proteins (NSPs) in ORF1ab, structure proteins, and accessory open reading frames (ORFs) among sarbecovirus isolates were analyzed (Fig. 3B, Tables S6 and S7).

When SARS-CoV and SARS-CoV-2 genomes are used as the query separately, to conduct the homology analysis, all known sarbecoviruses can be mainly divided into a SARSr-CoV branch (L1 in the MCC tree) and a SC2r-CoV branch (L2 in the MCC tree).

 Six newly identified isolates (YN2020B-G) collected from the southern border region of Honghe Prefecture in Yunnan share 95.8% nt identity with SARS-CoV at the whole-genome level, the highest identity with SARS-CoV among all known bat CoVs. The S genes of YN2020B-G are also highly similar to SARS-CoV, with 93.3% nt identity. We did not detect bat viruses highly related to SC2r-CoVs (L2-IP), despite the large scale of the sampling.

### Identification of recombinant lineage between SARSr-CoV and SC2r-CoV

Of note, four isolates, HN2021A, HN2021B, HN2021G and YN2021, identified from *R. pusillus* in Hunan and Yunnan provinces, together with previously discovered PrC31, ZXC21 and ZC45 [26,27], formed a new lineage, in which part of the viral genome, from NSP7 or NSP9 to NSP15, shows higher similarity to SARS-CoV, with 89.8%–97.8% nt identities, whereas the rest of the genome is more homologous to that of SARS-CoV-2, with 87.9%–92.5% nt identities. We named the new lineage L-R (R stands for recombination). The S genes of L-R are least conserved and showed only ∼73.0% nt identity with SARS-CoV and ∼75% nt identity with SARS-CoV-2, respectively (Fig. 3B and Table S7).

**Figure 4.**
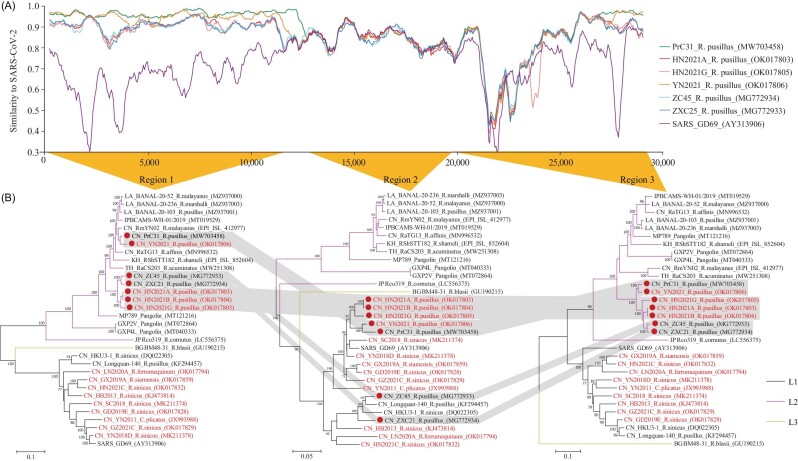
Recombination analysis for strains of L-R. (A) Similarity plot analysis. The full-length genome sequence of SARS-CoV-2 was used as a query sequence, and SARS-CoV, HN2021A, HN2021G, YN2021, PrC31, ZXC21 and ZC45 as reference sequences. (B) Maximum likelihood phylogenetic trees based on the three regions of the recombinant lineage alignment. Nucleotide positions for phylogenetic inference are 1–11 571 (region 1), 11 572–20 431 (region 2) and 20 891–29 899 (region 3). Numbers at internal nodes indicate bootstrap percentages, and gray-shaded regions show sequences exhibiting phylogenetic incongruence along the genome.

The extent of L-R recombination history can be illustrated by three phylogenetic trees inferred from two main breakpoints (Fig. 4). Ancestral recombination events divided the genome of L-R strains into SARSr-CoV-related region 2 and SC2r-CoV-related regions 1 and 3. When the first 11 kb and last 10 kb of the genomes were used to construct the tree, L-R could be phylogenetically clustered with L2-IP. When the middle part of the genomes between NSP9 and NSP15 was inspected, the L-R were clustered in L1. Because all L-R bat CoVs was only found in *R. pusillus*, the recombinant events likely happened in *R. pusillus*. Interestingly, the isolate Rc-o319, belonging to L2-JP when 440 nt RdRp sequences were used to perform the analysis (Fig. 3A), was detected in *R. cornutus* (a related species of *R. pusillus*) and has an ambivalent homology character, suggesting that Rc-o319 might have potential patterns of even more ancestral recombination with known lineages.

### The diversity and prevalence of bat sarbecoviruses in China

**Figure 5.**
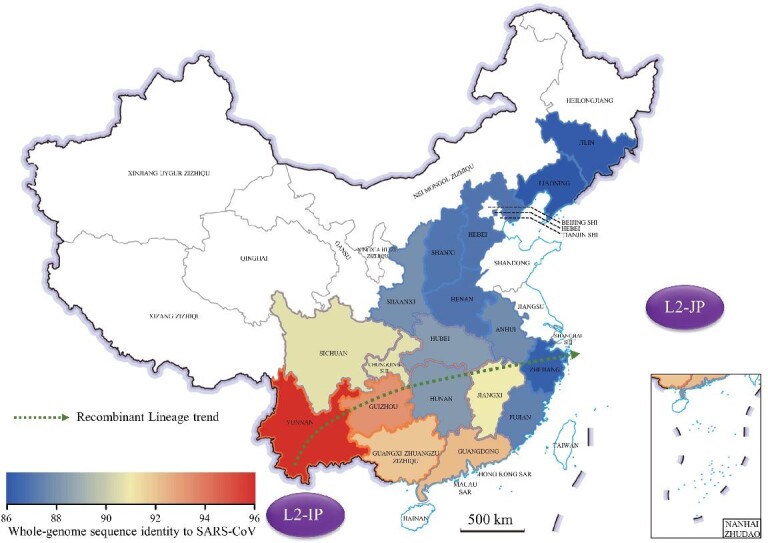
Map of highest genome similarity of bat sarbecoviruses in each province when compared with SARS-CoV. The colors of provincial boundaries are the same as the colors of L1 and L2 in Fig. 4, and the fill color of each province, ranging from blue to red, represents the genome similarity. The dotted arrows indicate the trend of similarity from higher to lower regions and link the recombinant strains between L2-IP and L2-JP. 审图号: GS京(2023)0922号.

By combining the genome homology with the divergent results of the MCC tree, the different genome similarities distributed in each province were illustrated (Fig. 5). A gross association between the gradual reduction in viral genome diversity and similarity of bat SARSr-CoVs with SARS-CoV, and the geographic direction from southwest China to northeast, can be found. The tendency grossly matches the possible spread directions of L1 in Fig. 2, with YN2020B-G sharing the highest genome sequence identity (95.8%) with SARS-CoV among all known bat CoVs. YN2020B-G were found in the southern border region of Honghe Prefecture in Yunnan province, raising the question of whether there is a bat SARSr-CoV that shares an even higher sequence identity with SARS-CoV further south of Yunnan.

Similarly, the L-R strains in *R. pusillus* also showed a similar trend with decrease in genome identity to SARS-CoV-2 from southwest to northeast (genome identity was reduced from 91.0%, 90.1%, 88.1%, 88%, 87.8%, to 87.7% nt and finally linked SC2r-CoVs in L2-IP (95.2% nt identity) and L2-JP (79.3% nt identity)) along limited survey sites when the SARS-CoV-2 genome was used as the query (Fig. 5).

### Characterization of S gene and receptor binding motif

**Figure 6.**
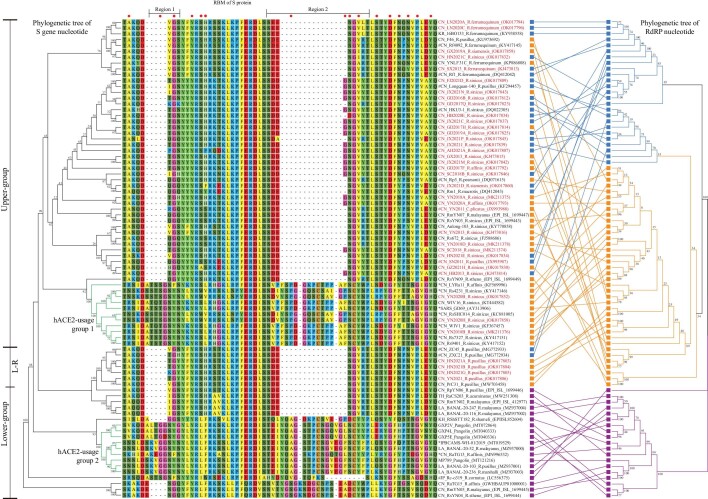
Phylogenetic trees based on S gene (left) and RdRp gene (right) are connected by the amino acid sequences of the RBM of sarbecoviruses. CoVs that have been confirmed or predicted to use hACE2 are shown in a green branch line, and those that have been shown to not use hACE2 are shown in a black branch line. All CoVs obtained in this study are labeled in red font. Amino acid numbering of RBMs is relative to SARS-CoV-2. L1.1 is labeled in blue, L1.2 is labeled in orange, L2 is labeled in violet.

The CoV S proteins bind the host receptors and play an essential role in virus entry. SARS-CoV, SARS-CoV-2 and several SARSr-CoVs and SC2r-CoVs use hACE2 as the entry receptor [28,29]. Phylogenetic analysis of S genes and sequence alignment of receptor binding motifs (RBMs) of different bat CoVs were performed (Fig. 6). All the CoVs in the RdRp-based L1 lineage were regrouped. The presence of the Upper-Group was consistent with the foregoing homology heatmap that showed obvious gene exchange between L1.1 and L1.2. Most members in this group have broad host species and do not bind hACE2 because of deletions in the two key regions of RBM, consistent with previous reports (labeled with #) [30]. The hACE2-usage-group-1 with intact RBM has relatively narrow host and location ranges in Yunnan province, and all were found in *R. sinicus* with the exception of one in *R. affinis*. Their capability of using hACE2 as the entry receptor has been experimentally verified among six CoVs (labeled with *) [10,29–31]. The L-R group also failed to bind hACE2 because of the presence of two deletions in the key regions of RBM.

All of the Lower-Group came from L2 and showed a more diverse deletion pattern in the RBM than the three groups described above. The hACE2-usage-group-2 had an intact RBM. It is worth noting that BANAL-20-52 from *R. malayanus* in Laos and MP789 from the Malayan pangolin had an almost identical RBM to SARS-CoV-2, except for residue 498 (Q498 in SARS-CoV-2, H498 in BANAL-20-52 and MP789). While the RBMs of RpYN06 and RmYN02 from the southern border region of Yunnan and RaCS203 from Thailand also had deletions in the two key regions, the RBMs of RShSTT182 and RshSTT200 from Cambodian *R. shameli* only had a 4 aa deletion in region 1, and the RBMs of Rc-o319 from Japanese *R. cornutus* only had a 9 aa deletion in region 2. Strikingly, although the S protein of RsYN04 also has a deletion in region 1 of the RBM, it can still weakly bind to hACE2 [21].

## DISCUSSION

Bat CoVs have high frequencies of recombination throughout the genome, making their cross-species transmission relatively easy and adaptation to new hosts rapid [32–34]. The discovery of bat SARSr-CoVs [7,9,11,35,36], particularly those using hACE2 as the entry receptor [10], highlights bats as the origin of SARS-CoV. Recently, several SC2r-CoVs were also detected in bats and some of them could also use hACE2 as the entry receptor [15–17,21]. Therefore, bats were also considered as the origin of SARS-CoV-2.

By the collection of >17 000 samples from 29 provinces between 2010 and 2021, we expanded the overall bat sample size to ∼50 000 in China. We not only covered most known geographic hotspots for bat CoVs, but also expanded into some new areas in China that have never been explored before, such as Hunan, Jiangxi, Fujian and Liaoning provinces. A large number of new SARSr-CoVs were detected, and some bat species, such as *R. luctus* and *R. siamensis*, were found to contain sarbecovirus for the first time. Colonization of diverse SARSr-CoVs was revealed in four *Rhinolophus* species: *R. sinicus, R. ferrumequinum, R. pusillus and R. affinis*. These four species tend to gregariously roost in groups, in caves with a relatively large population, which might facilitate active circulation and frequent recombination of such CoVs. Other accidentally related *Rhinolophus* species, such as *R. macrotis, R. siamensis* and *R. luctus*, always exist in small populations or even in pairs or individuals, which might make it difficult to keep SARSr-CoV circulating intraspecies. Although Hipposideridae bats, such as *H. armiger* and *H. pratti*, have a large population size and extensive habitat area in China, SARSr-CoV is rarely detected in these species. We speculate that these species may be contaminated or transiently infected by SARSr-CoV because many of them often share roosts with *Rhinolophus* species in the same cave, and they may not have the functional receptor(s) or required protease(s) for various sarbecoviruses. This warrants further investigation.

After providing a macroscopic view for bat CoVs, we find that the distribution pattern of the bat sarbecoviruses in China appears to have a geographic trend from southwest to northeast/east of China, with a gradual decrease in virus diversity across L2 and L1 lineages and their sublineages. Once a new lineage is established after differentiation, it might only spread in its own limited area; whether this phenomenon results from limited migration range of the hosts remains to be determined. Of note, this trend is also closely correlated with the degree of identity of genome sequences of SARSr-CoVs with SARS-CoV, higher genome identity in the southwest, and lower genome identity in the northeast/east of China. If this trend were also true outside China, the fact that YN2020B-G shares the highest sequence identity with SARS-CoV among all known bat SARSr-CoVs and was found near China's southwestern border would raise another important question about whether there would be any SARSr-CoVs sharing an even higher identity with SARS-CoV further south, even outside of China.

Several SC2r-CoVs, including RaTG13, RmYN02 and RpYN06, were found in bats from Tongguan and Xishuangbanna, Yunnan Province [15,16,21]. However, we failed to find any SC2r-CoVs, despite the fact that we collected 17 504 samples in 66 different bat species throughout significant parts of China between 2010–2021, including the border places where RaTG13, RmYN02 and RpYN06 were found, and our samples also included thousands of specimens from several *Rhinolophus* species known to harbor SC2r-CoVs. This result suggests that circulation of SC2r-CoVs in bats in China might be limited, and only present in a few isolated locations near the southern border of China, such as RmYN02 and RpYN06 in Xishuangbanna Tropical Botanical Garden. As for RaTG13, this virus was not found again in Tongguan cave and its surrounding areas despite repeated sampling conducted by Dr. Shi's group and ours [37–40]. Recently, SC2r-CoVs were found in *R. shameli, R. acuminatus* and *R. marshalli*, which mainly inhabit SEA [17,19,20]. *Rhinolophus malayanus*, the host bat for RmYN02 and BANAL-20-52, is also an endemic species in SEA and was firstly recorded as a northward-migrating species in the southwestern frontier of China in 2015 [41]. The *R. pusillus* and *R. affinis* bats, in which RpYN06 and RaTG13 were found, respectively, are also commonly found in China, SEA and South Asia. Whether there might be more SC2r-CoVs actively circulating in *Rhinolophus* species warrants further investigation [42].

To date, the bat CoVs sharing the highest nt identities with SARS-CoV and SARS-CoV-2 are YN2020B and BANAL-20-52, and they only show 95.8% and 96.8% nucleotide sequence identity with SARS-CoV and SARS-CoV-2, respectively [10,15,19]. The hypothesis that bats act as the direct natural reservoir of SARS-CoV and SARS-CoV-2 is challenged by the remaining genetic gaps. The lineages composed by different CoVs have undergone independent evolution ever since the estimated divergence events occurred (∼60–70 years ago for SARS-CoV and its closely related SARSr-CoVs, and ∼40–70 years ago for SARS-CoV-2 and RaTG13), indicating that none of the present known bat CoVs could serve as the immediate precursors for these two hCoVs [24]. Further CoV surveys are urgently needed to identify the potential ancestor, and should be expanded to the regions where susceptible *Rhinolophus* species are present, especially places with under sampling [42].

Animal spillover, in which the virus spills over from bats to animal-intermediate-host, then to humans, has been proposed as another plausible theory [1]. Zoonotic transmission among different species might accelerate the evolving rate of viral genomes, leading to the homology gap in viral genomes. Potential intermediate hosts may include Malayan pangolins, rabbits, ferrets, foxes and raccoon dogs, because their ACE2s can bind to S of SARS-CoV and SARS-CoV-2 and facilitate virus entry [29,43–45]. The S-based phylogenetic analysis showed that the S genes of L2 and L1.2 are more diverse than those of L1.1. Further analysis reveals that the RBMs of L2 in the southwestern border of China and Indochina Peninsula are the most diverse, suggesting that they might have more flexibility to adapt to new hosts via recombination. Considering that almost identical RBMs of SARS-CoV-2 were found in SC2r-CoVs of *Rhinolophus* bats in Laos and smuggled Malayan pangolins, the possible transmission route from bat to pangolin, then to human, warrants further investigation. Recently, this hypothesis was, in part, further supported by the detection of SARS-CoV-2 neutralizing antibodies in bats and Malayan pangolins in SEA [17].

The finding of recombinant isolates sheds light on the natural origins of human sarbecoviruses [46,47]. The detection of *R.-pusillus*-related L-R reveals that complicated recombinant events have happened between SARSr-CoV-related L1 and SC2r-CoV-related L2-IP during virus–host co-evolution in this bat species. The ancestor of L-R may come from an L2-IP strain that exchanged the middle genome fragment with SARSr-CoV. This recombinant lineage firstly colonized in *R. pusillus* when entering the southwest border of China and then spread to the northeast with an independent evolution clade along the gradual population dispersal of *R. pusillus*. Further sequence analysis of RmYN02, RpYN06 and L-R strains reveals that the ancestor of L-R might originate from an L2-IP recombining with SARSr-CoV. Although the risk to humans from L-R strains remains low because of their hACE2 unusable feature, we should not underestimate their cross-species abilities, through recombination, to obtain new S genes.

The origin of SARS-CoV-2 has been highly controversial. Recently, several studies showed that there might have been imperceptible SARS-CoV-2 circulation outside China before the major outbreak in Wuhan and suggested a reasonable twin-beginning interpretation [48–51]. Our bat CoV data in conjunction with studies tracking the spread of SARS-CoV-2 in human populations provide compelling rationales for searching for its origin beyond the mainland of China and even in SEA [51–53]. An extensive animal CoV survey further south and southwest of China is needed to find closer ancestors of human sarbecoviruses. Furthermore, bats outside of pan-SEA regions may also deserve attention, according to the epidemiological analyses of SARS-CoV-2 [51–53].

While this manuscript has been under revision, a News Feature has been published in the journal *Science*, questioning the validity of our result [54]. We would like to use a quote from R.A. Fisher, ‘let data speak for themselves’ [55], and we stand by our data. Furthermore, while there is no need for any authors to defend their unbiased attitude toward the survey design, we would like to point out that the surveys were conducted in three different periods 2010–2013 [25], 2016–2019 and 2020–2021 (see the first section of Results). As most of the surveys were done prior to the onset of COVID-19, the surveys could not have been influenced, consciously or subconsciously, by what has happened since December 2019. Scientists should have an open mind and focus on the data themselves. Investigation into the origin of SARS-CoV-2 is very complicated, and global collaboration, not politics, is urgently needed.

## MATERIALS AND METHODS

### Ethics approval and consent to participate

Animals were treated according to the guidelines of the Regulations for the Administration of Laboratory Animals (Decree No. 2 of the State Science and Technology Commission of the People's Republic of China, 1988). Sampling procedures were approved by the Ethics Committee of the Institute of Pathogen Biology, Chinese Academy of Medical Sciences and Peking Union Medical College (approval number: IPB EC20100415).

### Sample collection

With a combination of hand nets (for catching bats in a cave or building), mist nets (for sealing the exit of a cave or building) and harp traps (for catching bats in a forest or wood), bats were trapped in their natural habitat in karstic caves, woods, forests or abandoned buildings. Each bat was stored in a cotton bag. Pharyngeal and anal swab samples were collected from live bat individuals and then immersed in virus sampling tubes (Yocon, Beijing, China) containing maintenance medium, and temporarily stored at −20°C or in liquid nitrogen. Bats were released at the site of capture immediately after sampling. Samples were then transported to the laboratory and stored at −80°C. The identity of the captured species was initially determined by morphology by experienced field biologists and subsequently confirmed by barcoding of mitochondrial cytochrome b using patagium. The accurate locations of sampling sites were recorded by place names and GPS coordinates with latitude and longitude.

### Library construction and next-generation sequencing

Samples from the same bat species and from the adjacent sites were pooled by adding 1 ml from each maintenance medium sample into one new sample tube. The pooled samples were processed with a virus-particle-protected nucleic acid purification method as described previously [56]. The samples were homogenized in virus maintenance medium and subsequently filtered through a 0.45 μm polyvinylidene difluoride filter (Millipore, Germany). The filtered samples were then centrifuged at 150 000 × g for 3 h. The pellet was digested in a cocktail of DNase and RNase enzymes. The viral DNA and RNA were simultaneously isolated using a QIAmp MinElute Virus Spin Kit (Qiagen, USA). First-strand viral cDNA was synthesized using the primer K-8N and a Superscript IV system (Invitrogen, USA). The cDNA was converted into dsDNA by Klenow fragment (NEB, USA). Sequence-independent PCR amplification was conducted using primer K. The PCR products, which are from 300 to 2000 bps, were purified by magnetic beads (Beckman Coulter, USA). The purified products were then subjected to an Illumina HiSeq X Ten sequencer, for a paired-end read of 150 bp. The sequence reads were filtered using previously described criteria [57]. Clean data were generated after the adaptor sequence, primer K sequence, and low-quality reads were removed.

### Taxonomic assignment and genome assembly

Sequence-similarity-based taxonomic assignments were processed as described in our previous study [58]. Reads in clean data were aligned to sequences in the NCBI non-redundant nucleotide database and non-redundant protein database using BLASTx. The taxonomies of the aligned reads with the best BLAST scores (E score <10−5) were parsed and extracted by MEGAN6 [59]. Extracted coronavirus reads were assembled by megahit v1.2.9 with default parameters. Assembled contigs were used as reference sequences during PCR screening and sequencing.

### PCR screening and genome sequencing

CoV screening of pooled or individual samples was performed by amplifying a 440-bp fragment of the RdRp gene of all the α-CoVs and β-CoVs using conserved primer pairs as described previously [7,60–62]. Specific primers were designed from assembled contigs of sarbecovirus and their closest reference sequence identified by BLAST from Genbank. Each ORF in the viral genome was amplified with nested specific primers whose PCR product was nearly 1500 bp, and then sequenced with ABI3500 DNA analyzer (Applied Biosystems, USA). PCR products with low concentration, or that were generating heterogeneity in the sequencing chromatograms, were cloned into pMD-18T Vector (Takara) for sequencing.

### Phylogenetic and recombination analysis

MEGA7.0 was used to align nucleotide sequences and deduce amino acid sequences using the MUSCLE package and default parameters [63]. The best substitution model was then evaluated by the Model Selection package. Finally, we constructed a maximum-likelihood method using an appropriate model to process the phylogenetic analyses, with 1000 bootstrap replicates. Recombination among CoVs was detected with SimPlot software. All analyses were performed with a Kimura model, a window size of 200 bp and a step size of 20 bp.

### Bayesian analysis of molecular sequences

Partial RdRp sequences (∼440 nt) were used to reconstruct phylogenies and evolutionary hypotheses. They were performed using a Bayesian method implemented in BEAST v.1.10.4. A GTR + Gamma(4) + Invariant nucleotide substitution model, a strict clock model, and the constant size model as a coalescent tree were also selected for the analyses, which were run for 10 million steps with sampling at every 1000 states. The BEAGLE parallel computation library was used to enhance the speed of the likelihood calculations. Since the estimation of the most recent common ancestor may not be fit for partial RdRp sequences of CoVs, we did not add timing information and calibration in this analysis [64–66]. Finally, the resulting log file was checked using TRACER version 1.5 (http://tree.bio.ed.ac.uk/software/tracer) to confirm that all effective sample sizes were >200. TreeAnnotator was used to summarize posterior tree distributions and annotate the estimated values to a maximum clade credibility tree with a burn-in of 10%, which was visualized using FigTree v1.4.4.

### Homology analysis of all NSPs and structure proteins

Separate alignments for the main ORF and NSP genes were generated by MAFTT v.7.475. The sequence distance of 15 NSP genes belonging to ORF1a and ORF1ab, structure protein genes, and several accessory proteins, were analyzed by MegAlign (DNA Star package). Homology detection between each part of these complete genome strains was virtualized by heatmap made by TBtools v1.082 [67].

## DATA AVAILABILITY

All genome sequences that support the findings of this study have been deposited in GenBank with accession numbers OK017594 to OK017860. The barcoding data of species have been deposited in GenBank with accession numbers ON640659 to ON640727. The raw NGS data reported in this paper have been deposited in the Genome Sequence Archive in the National Genomics Data Center, China National Center for Bioinformation/Beijing Institute of Genomics, Chinese Academy of Sciences (GSA: CRA006652) at https://ngdc.cncb.ac.cn/gsa.

## Supplementary Material

nwac213_Supplemental_Files
