# Supplementary material for: A comprehensive survey of bat sarbecoviruses across China in relation to the origins of SARS-CoV and SARS-CoV-2
Source: Natl Sci Rev. 2022 Oct 11;10(6):nwac213. doi: 10.1093/nsr/nwac213 (PMC10325003; doi:10.1093/nsr/nwac213)
Supplement: nwac213_Supplemental_Files [file nwac213_supplemental_files.zip › Supplemental-figure legends.docx]

**Supplementary Fig. 1. Geographical distributions of other bat species concerned in this study.** The main distribution ranges of *R. luctus*, *R. paradoxolophus, R. siamensis, Cynopterus sphinx, Eonycteris spelaea, Eptesicus serotinus, Hipposideros cineraceus, Hipposideros larvatus, Hypsugo cadornae, Hypsugo pulveratus, Ia io, Megaderma lyra, Megaderma spasma, Miniopterus pusillus, Miniopterus schreibersii, Myotis petax, Myotis altarium, Myotis chinensis, Myotis fimbriatus, Myotis formosus, Myotis indochinensis, Myotis laniger, Myotis longipes, Myotis montivagus, Myotis nipalensis, Myotis ricketti, Myotis rufoniger, Myotis siligorensis, Nyctalus velutinus, Pipistrellus abramus, Pipistrellus ceylonicus, Pipistrellus pipistrellus, Rousettus leschenaultia, Scotophilus heathi, Scotophilus kuhlii, Taphozous melanopogon, Tylonycteris pachypus,* and *Tylonycteris robustula* are labeled in yellow color. Further information for these species can be found from the International Union for the Conservation of Nature (https://www.iucnredlist.org/).

**Supplementary Fig. 2.** **(A).** **Phylogenetic tree based on the partial RdRp (NSP12) sequences of CoVs of 200 pools.** **(B).** **Phylogenetic tree of sarbecoviruses, inferred from partial RdRp (NSP12) sequences of 146 individual samples.** Numbers at internal nodes of A&B indicate bootstrap percentages. All viruses found in this study are labeled in red. Details of the isolates of pool and individual samples are given in Extended Data Tables 4 and 5. **(C). Heatmap based on the normalized sequence reads of CoVs in each pooled sample.** The pool numbers are listed in the right text column. The color of the boxes, ranging from light to dark, represents the relative abundance of CoV-associated reads in each pool. The blue box represents α-CoV positive alone, the red box represents β-CoV positive alone, and the violet box represents α-CoV and β-CoV positive.
