# Supplementary figures and images for: A comprehensive survey of bat sarbecoviruses across China in relation to the origins of SARS-CoV and SARS-CoV-2
Source: Natl Sci Rev. 2022 Oct 11;10(6):nwac213. doi: 10.1093/nsr/nwac213 (PMC10325003; doi:10.1093/nsr/nwac213)

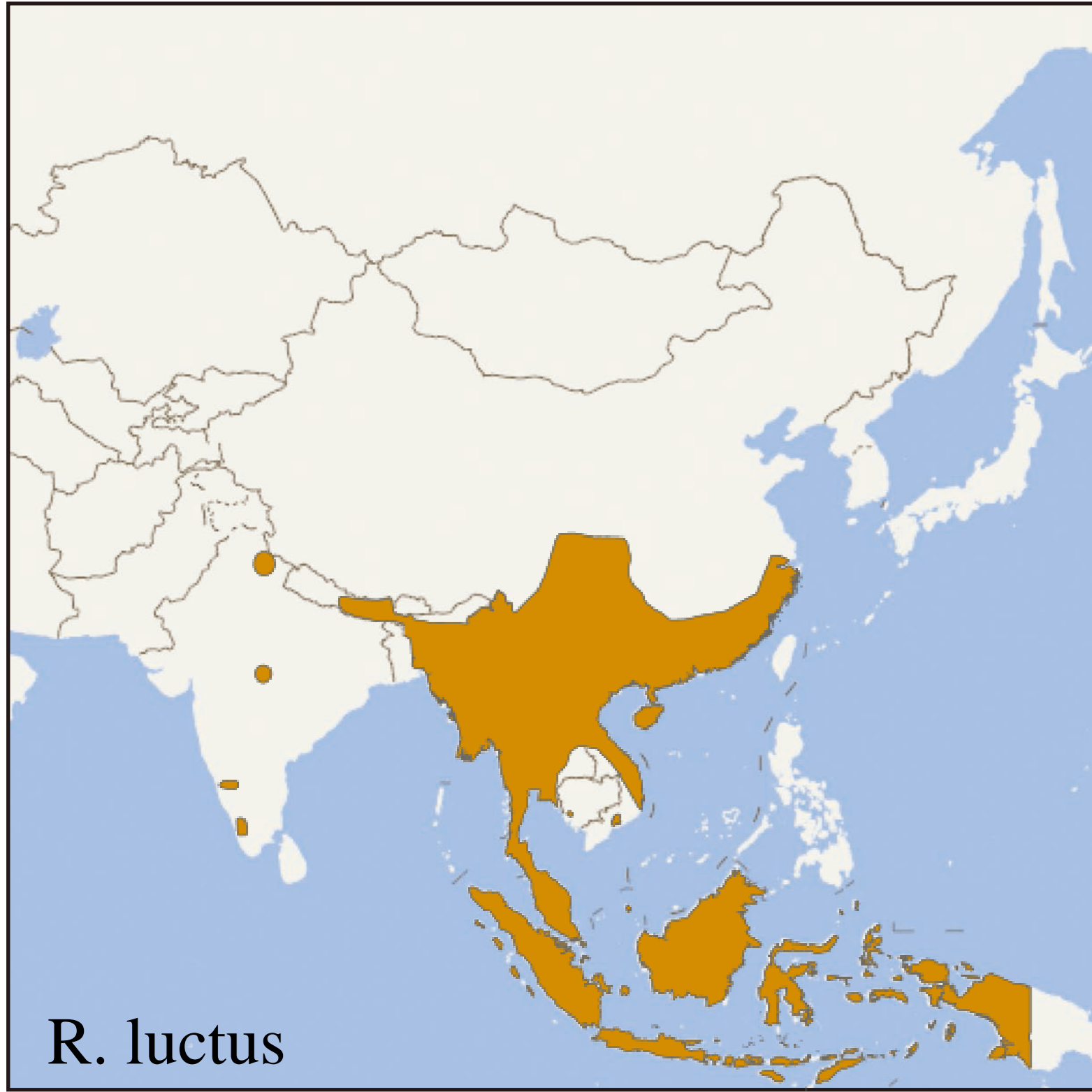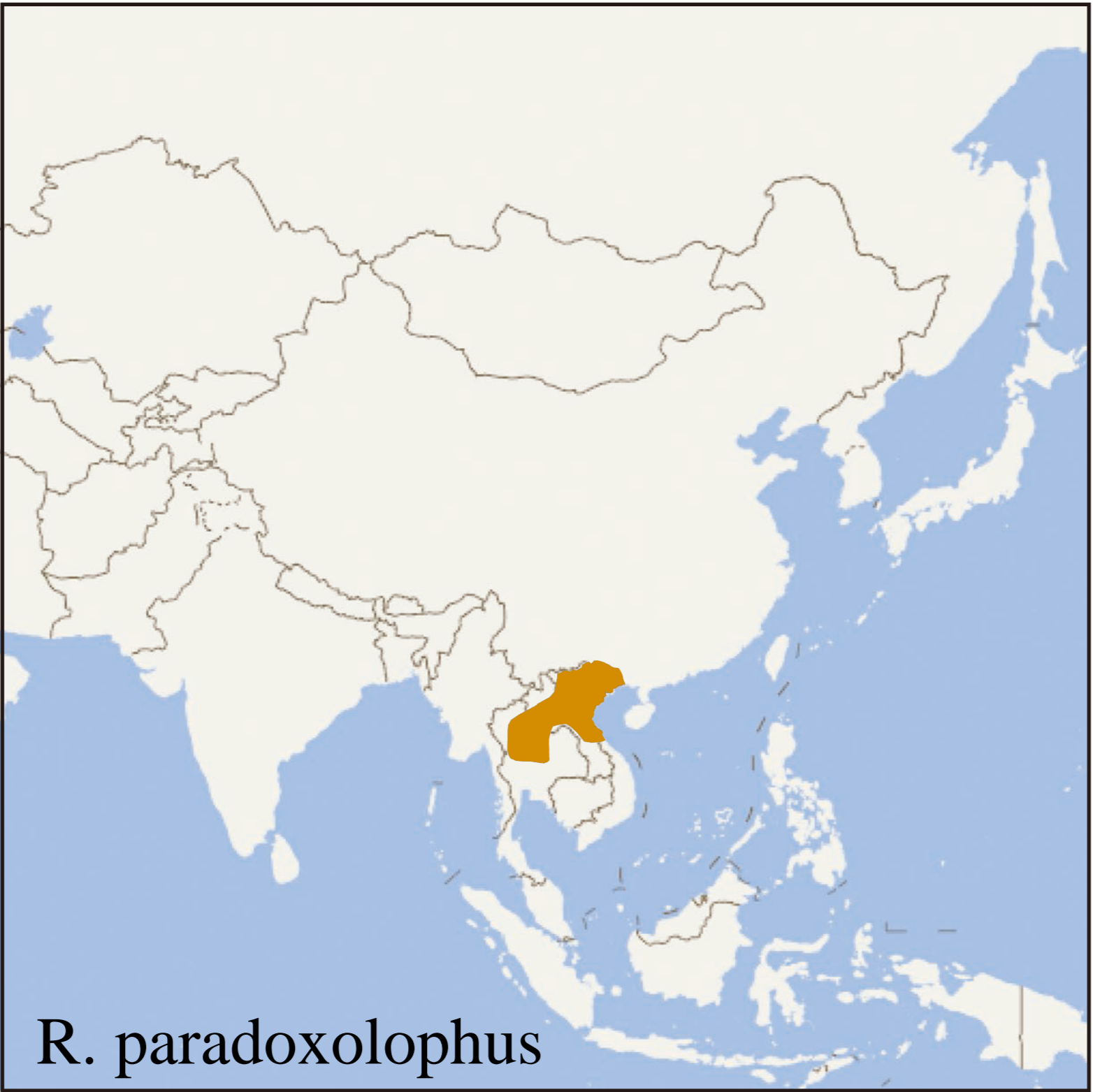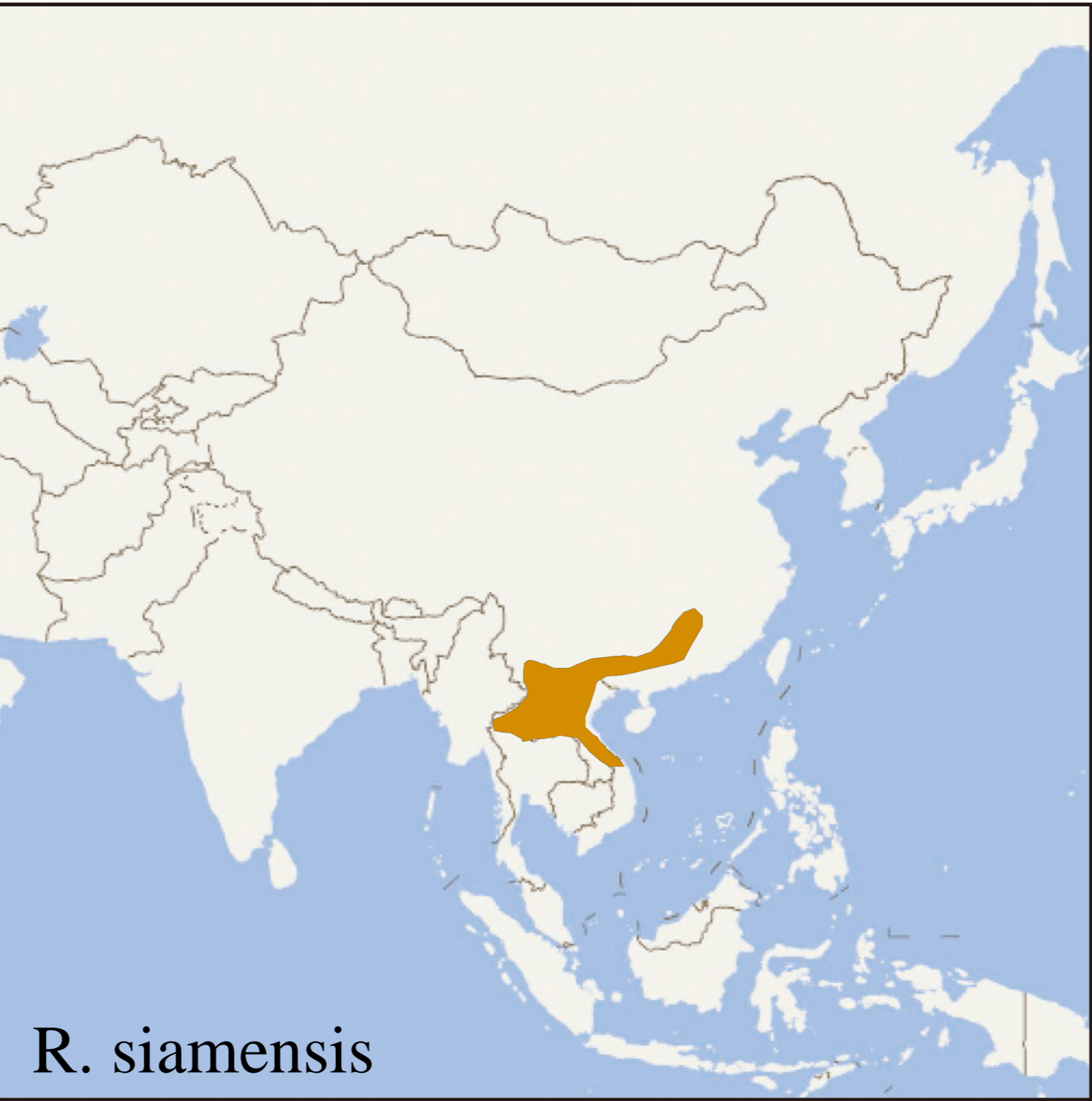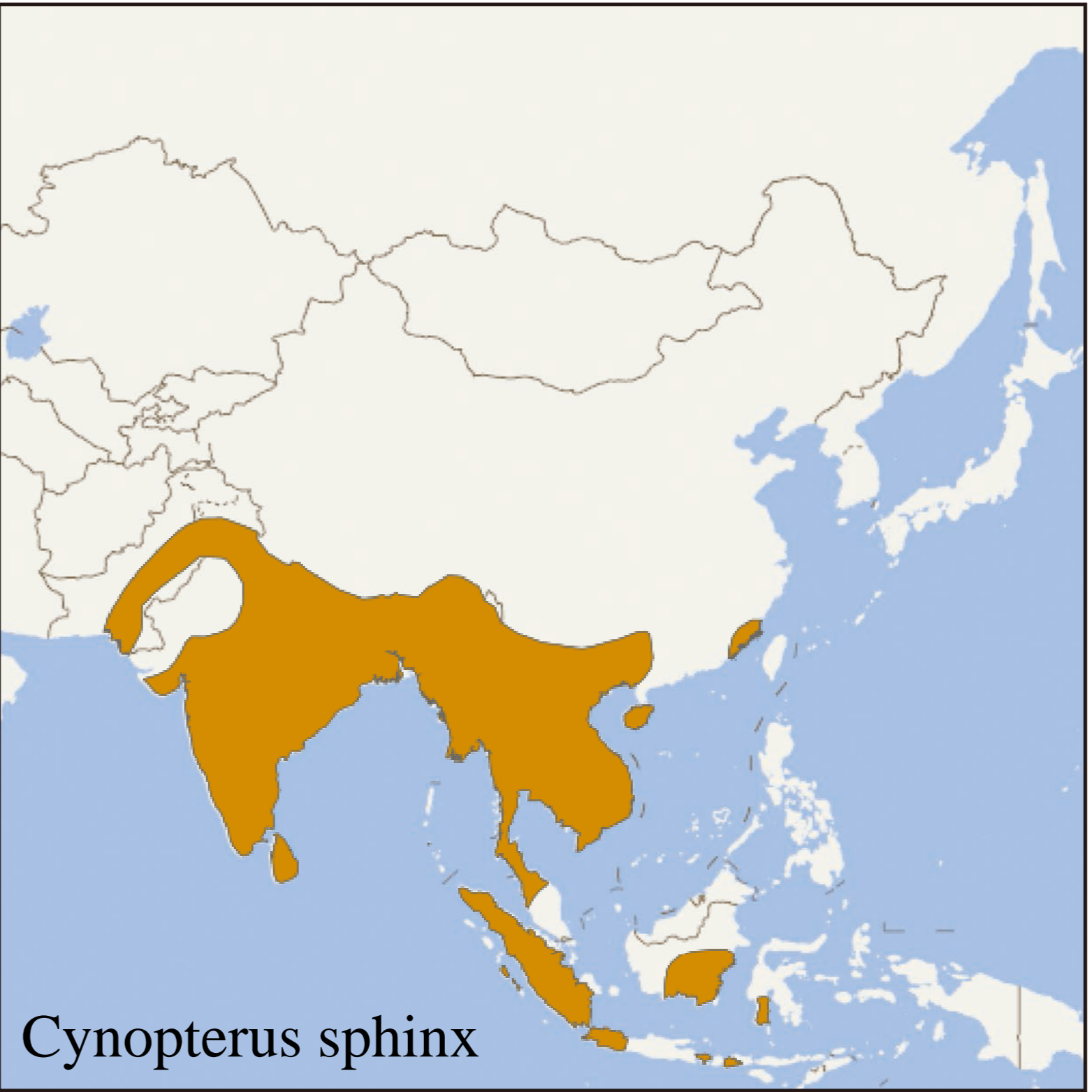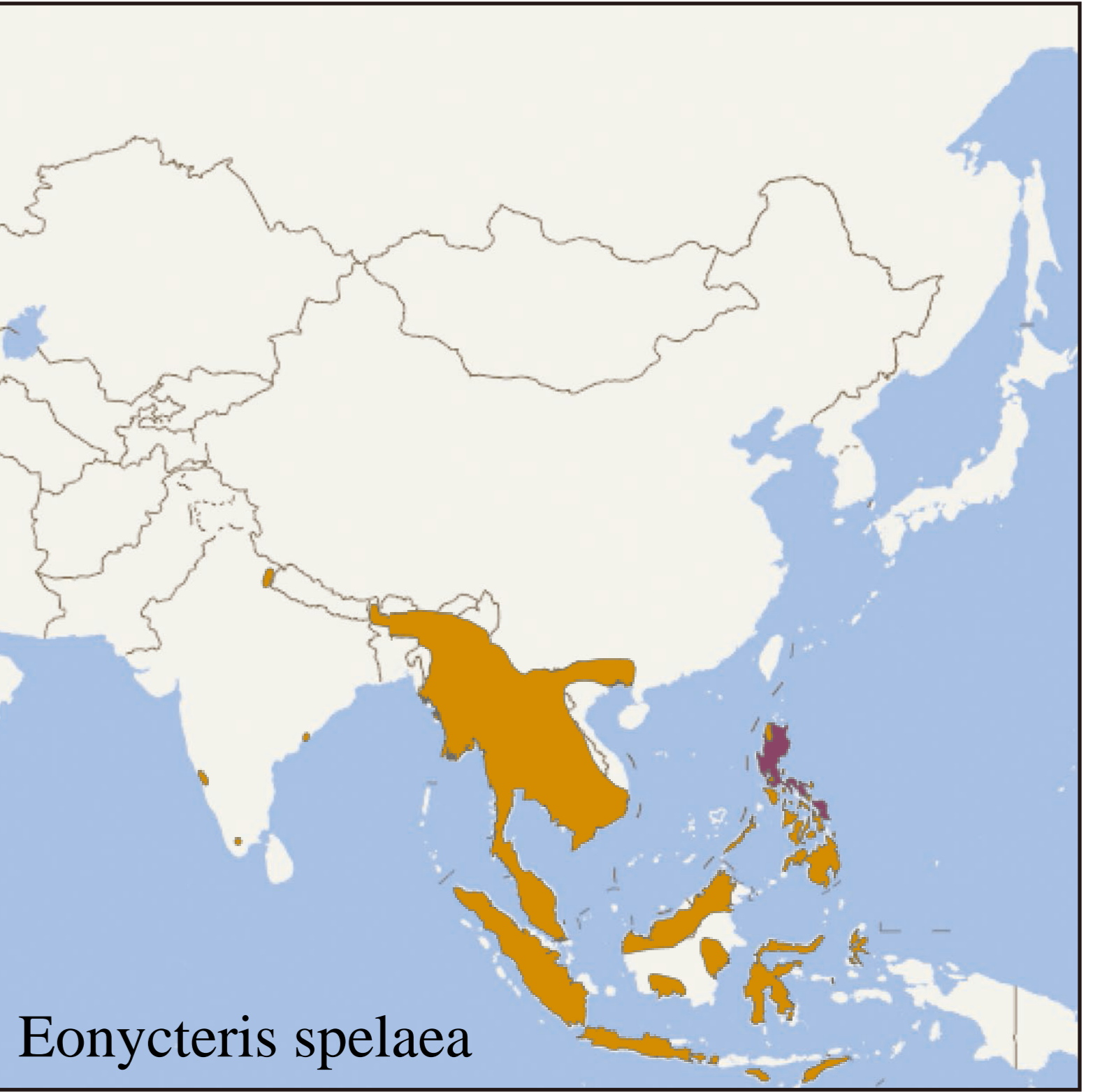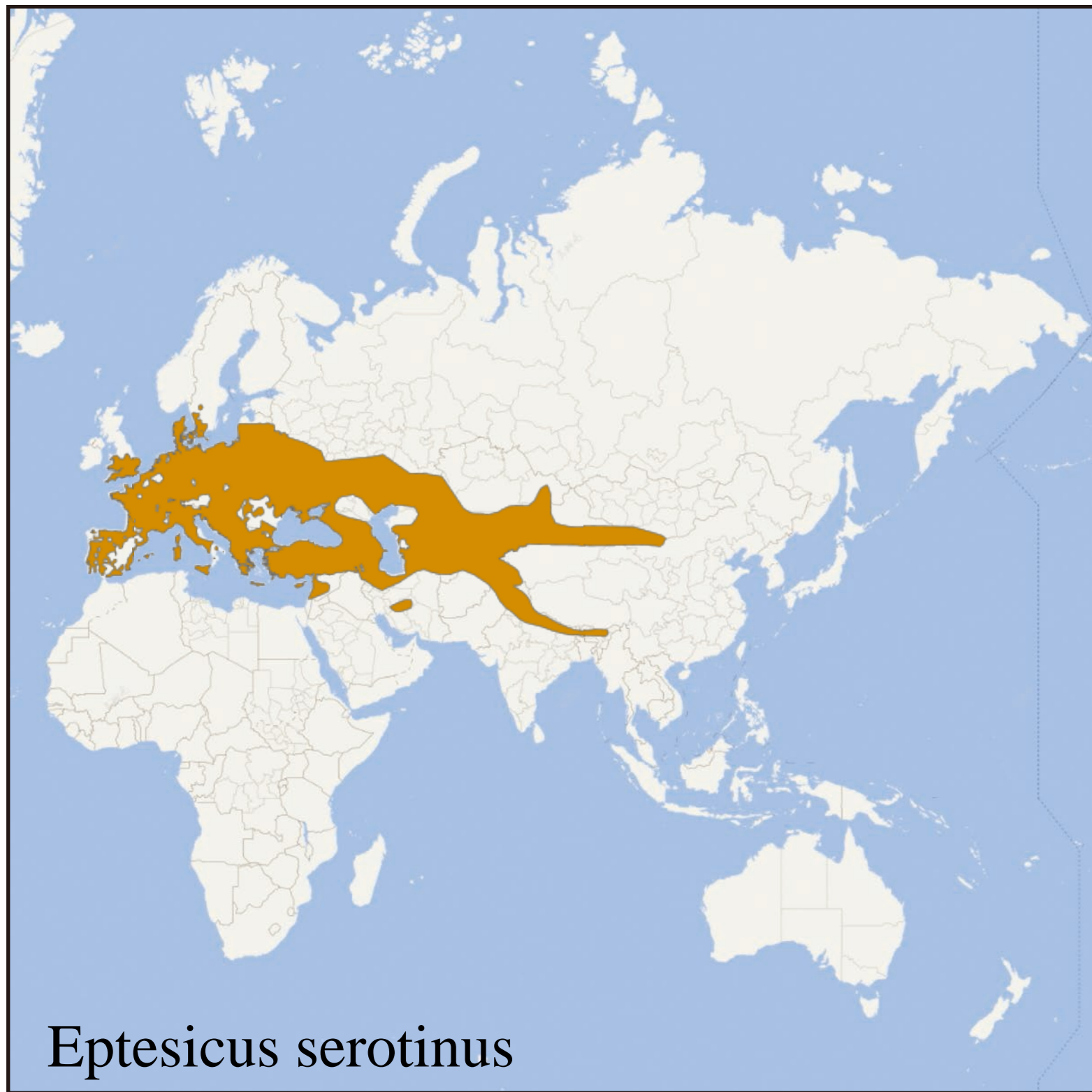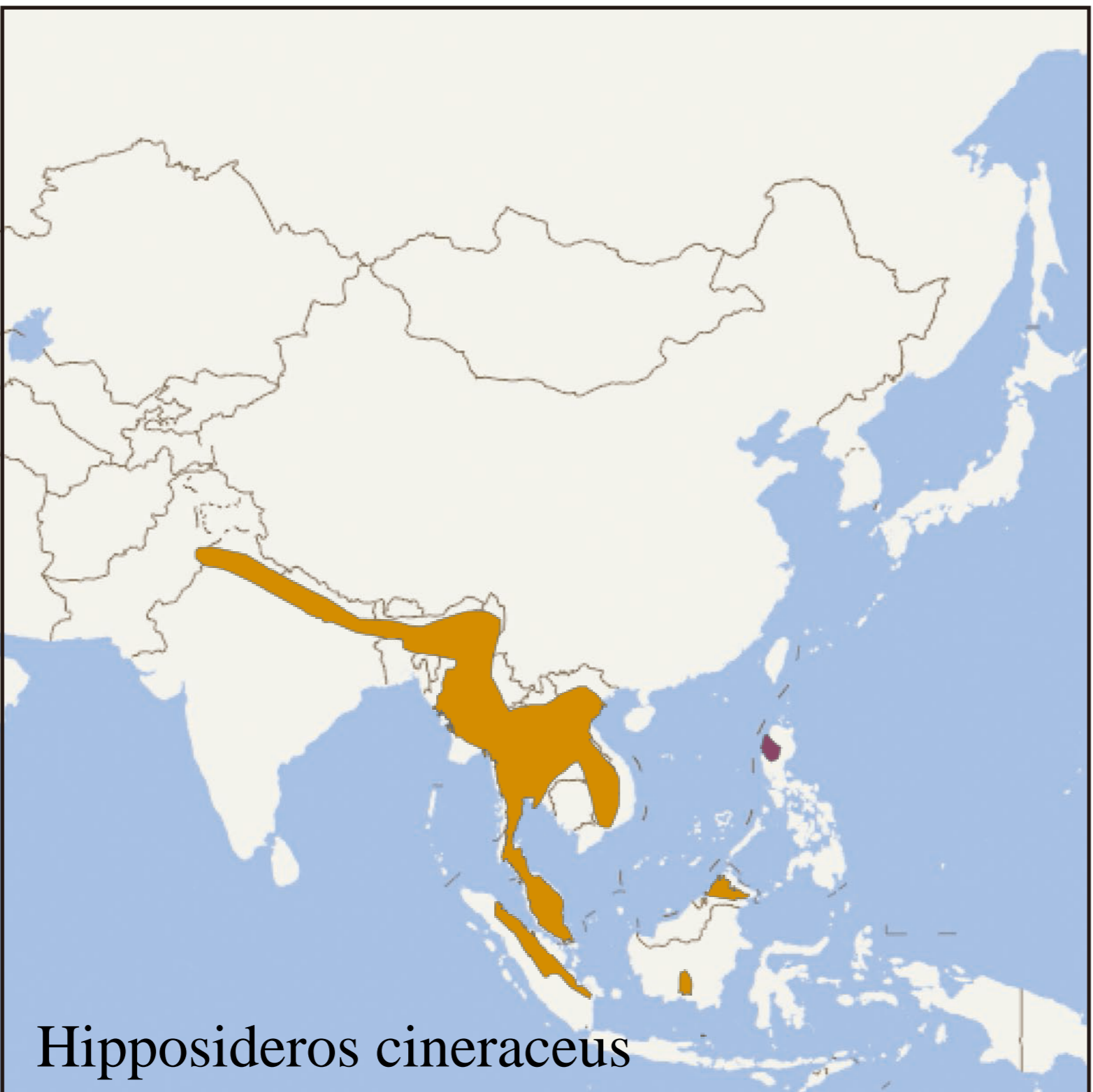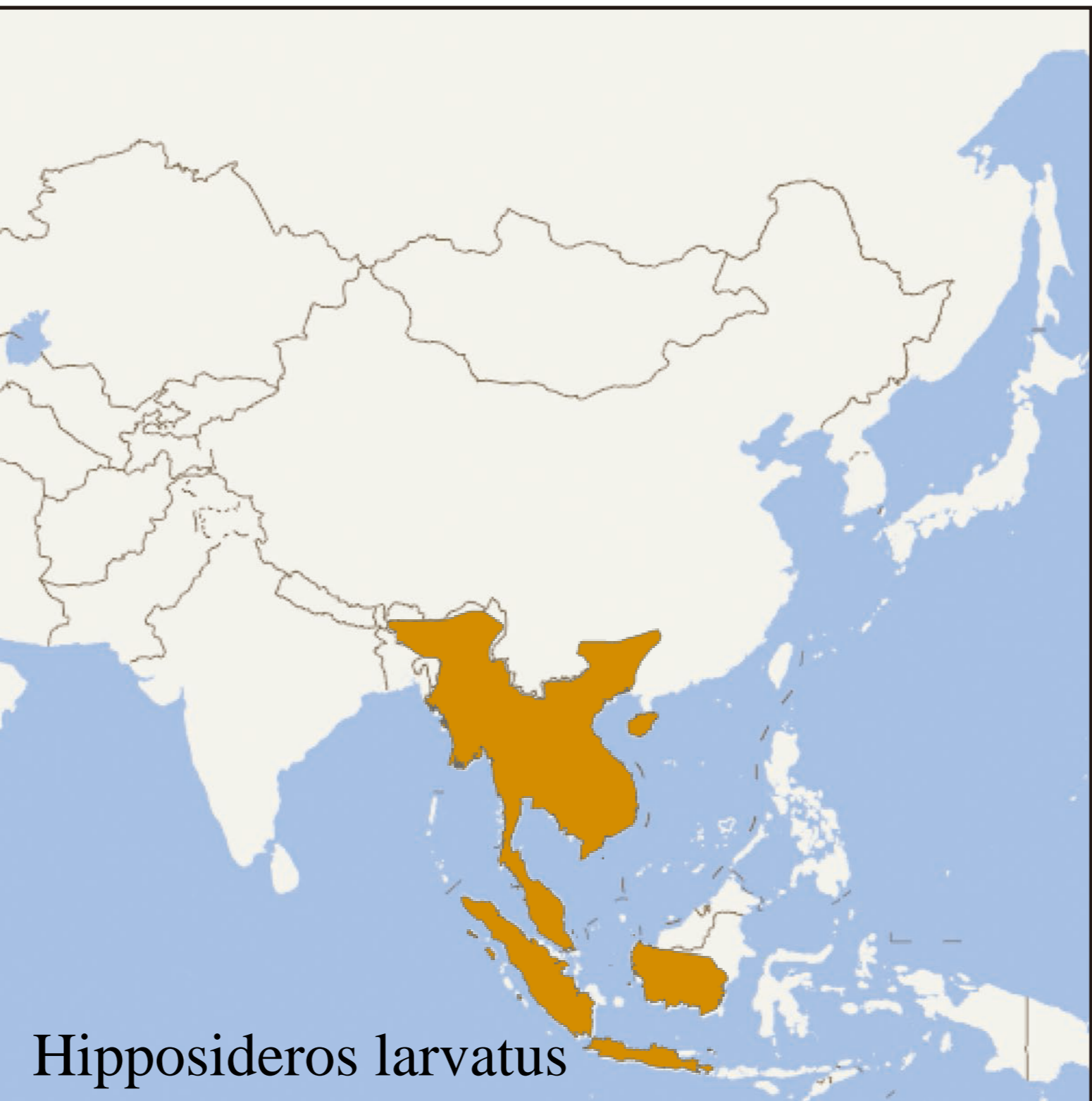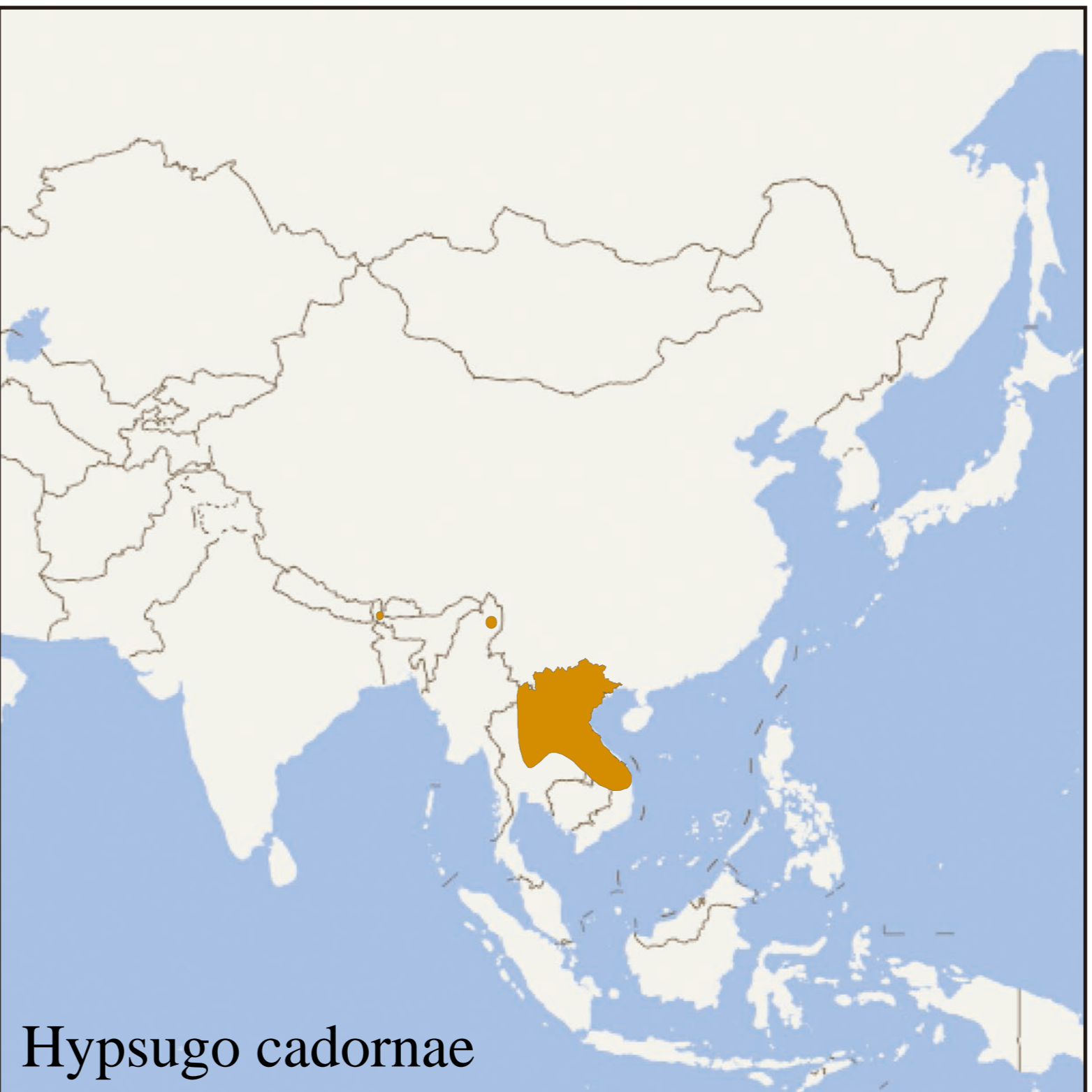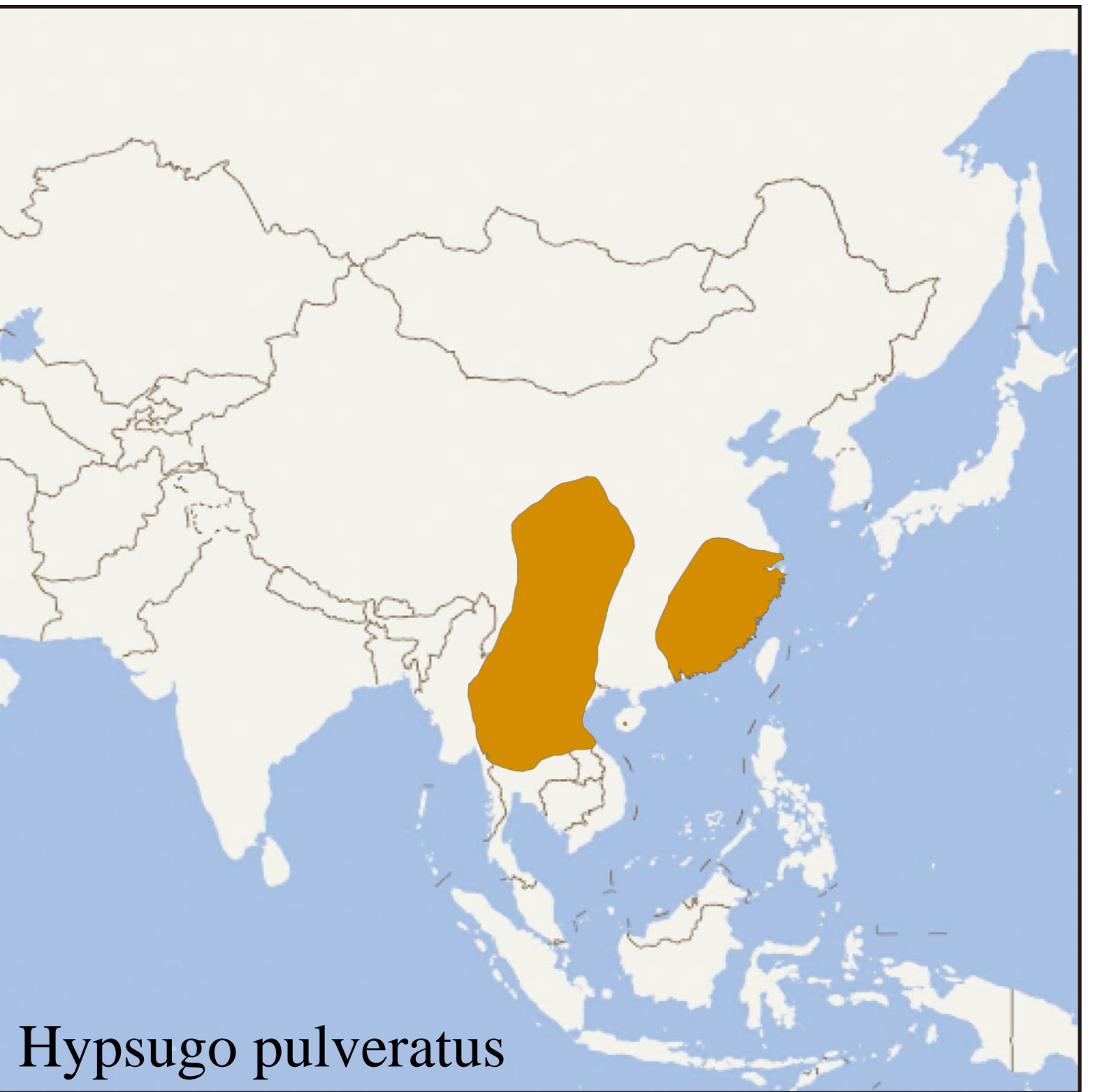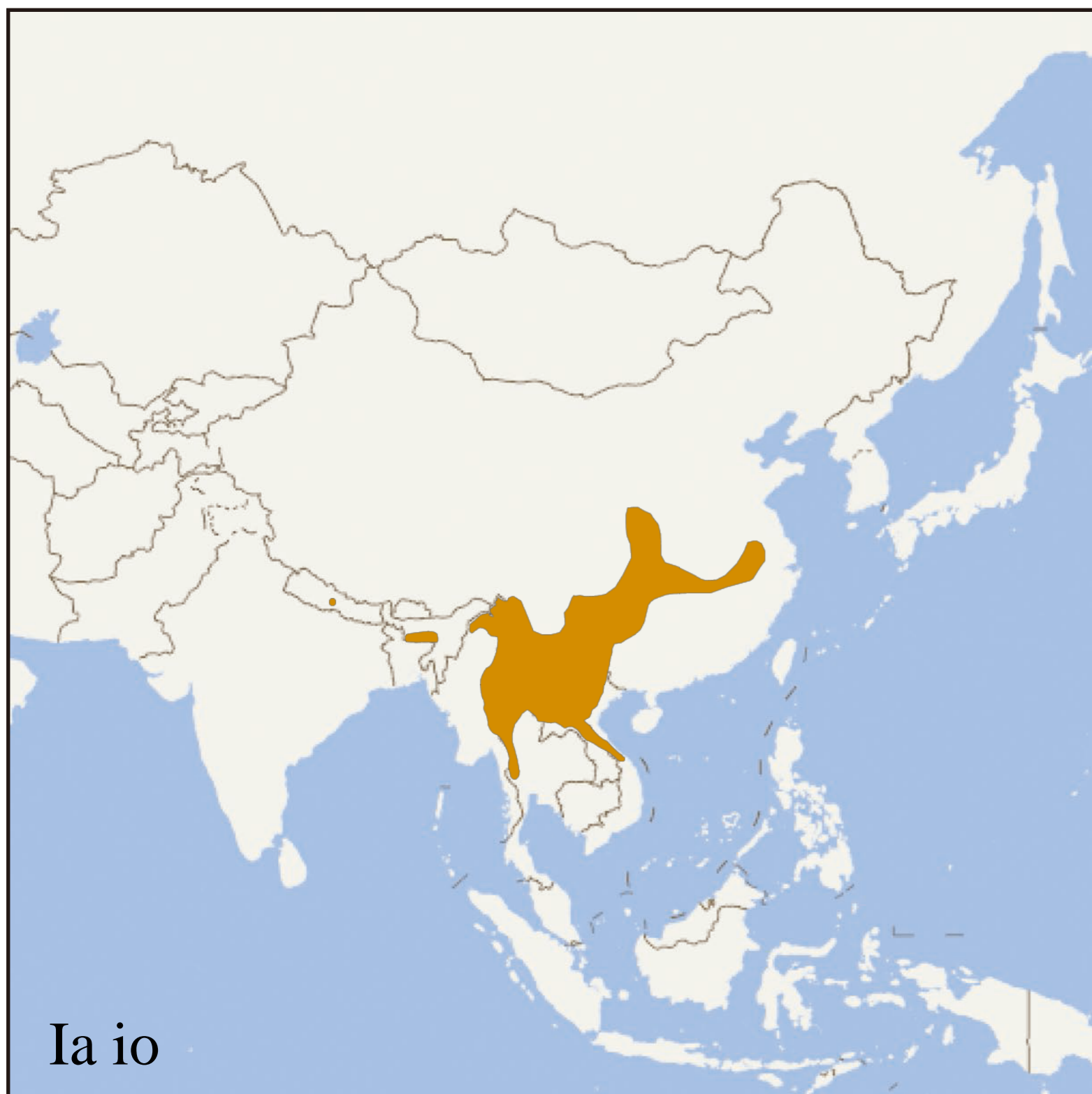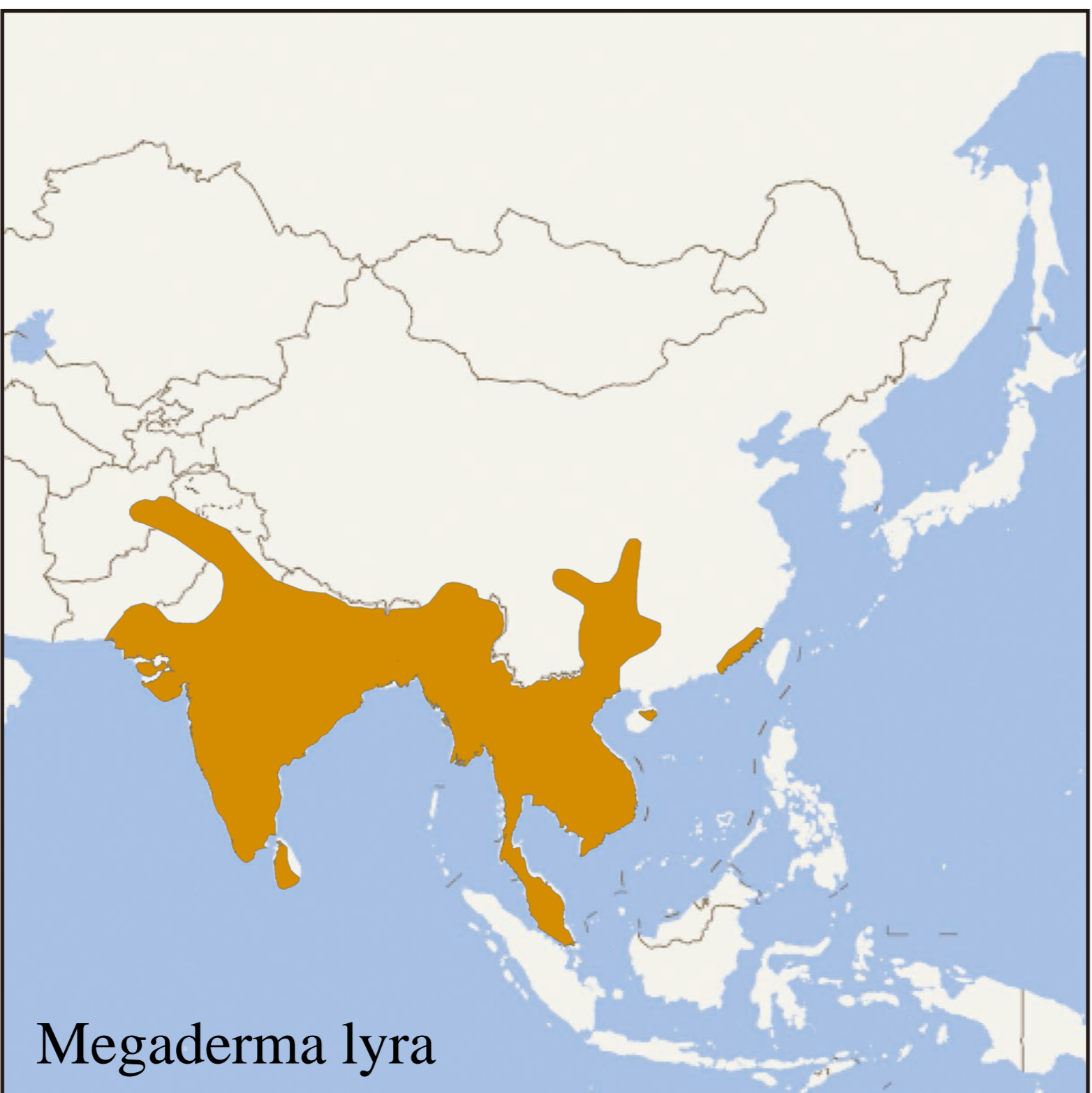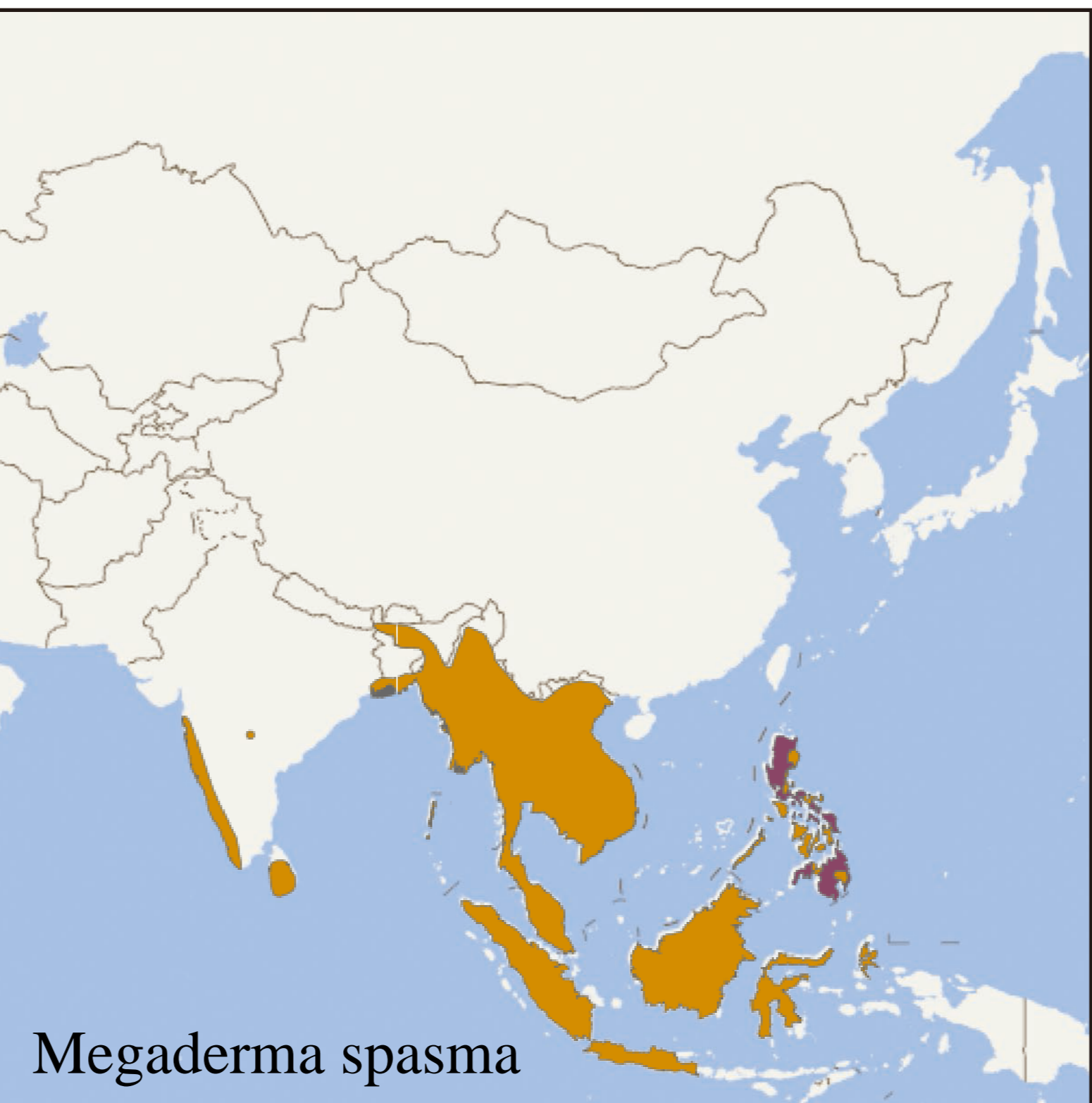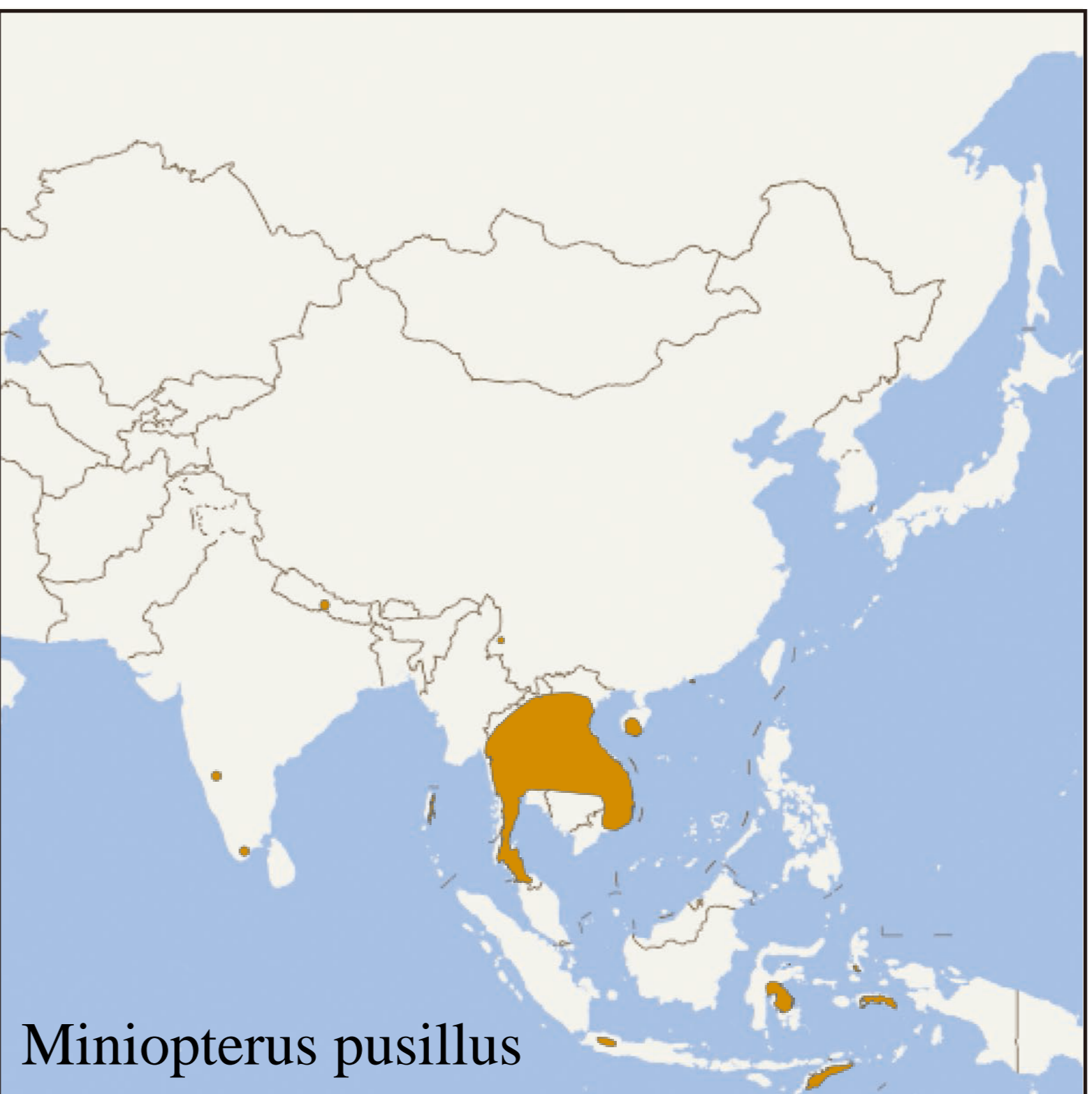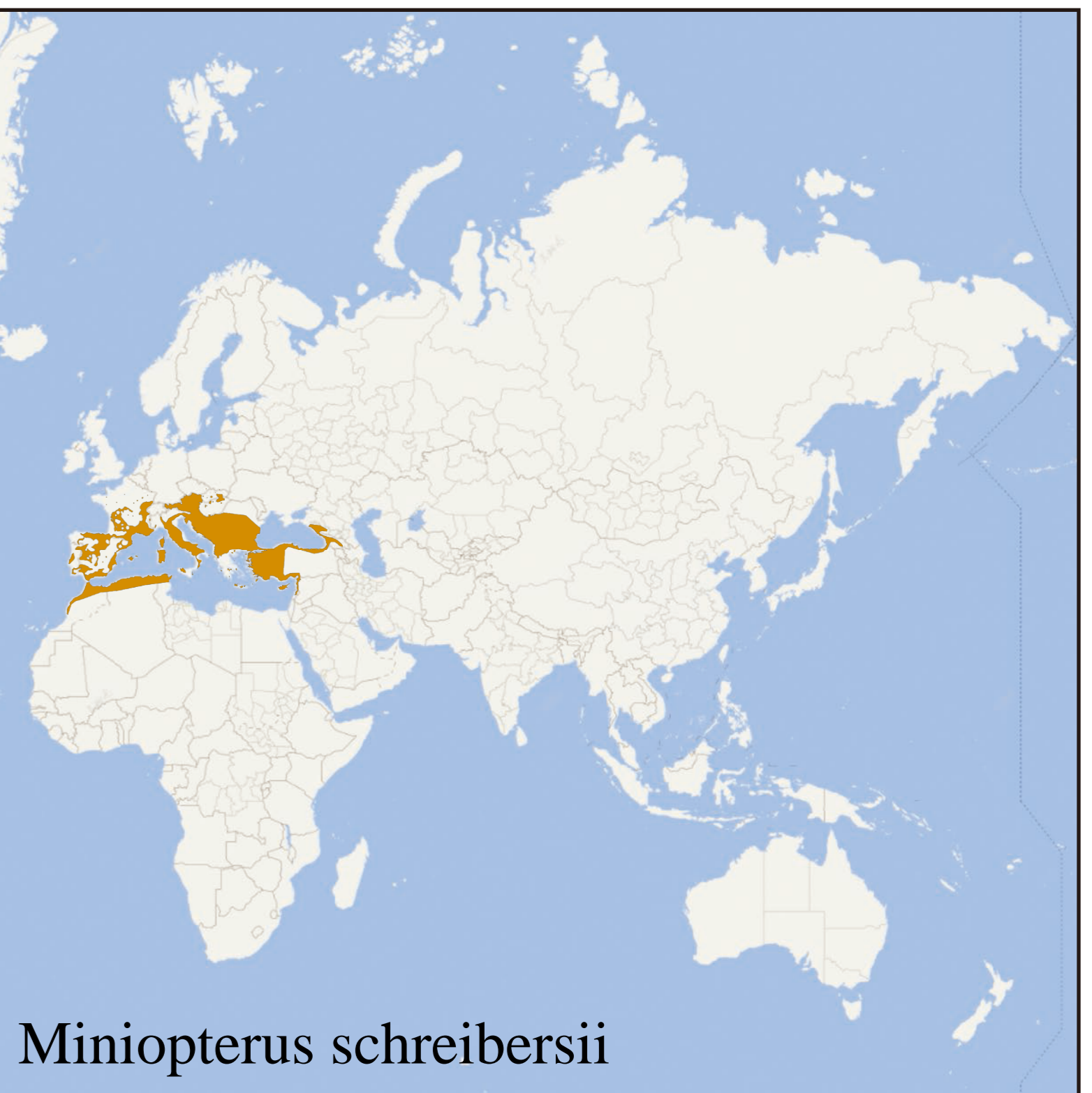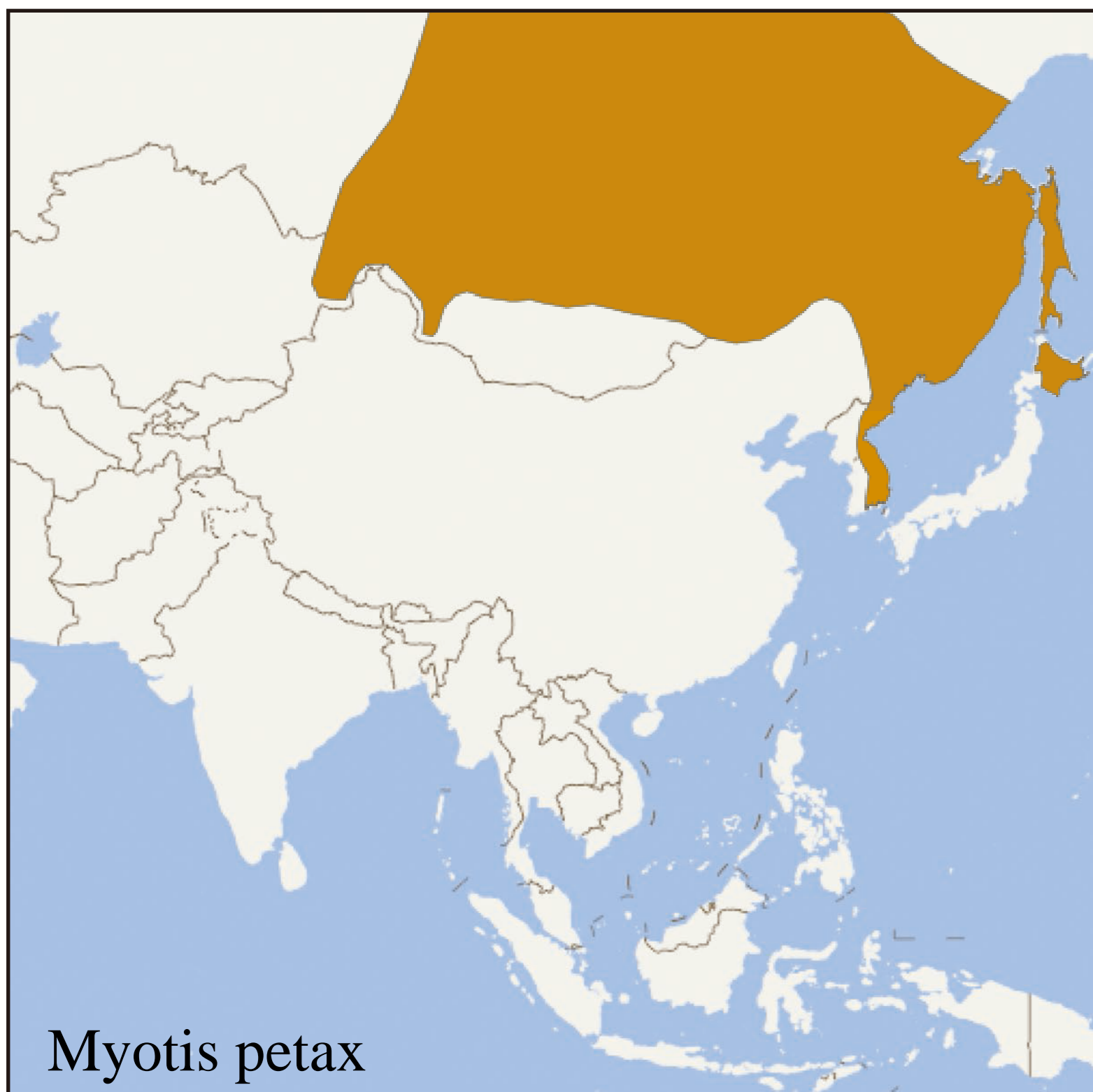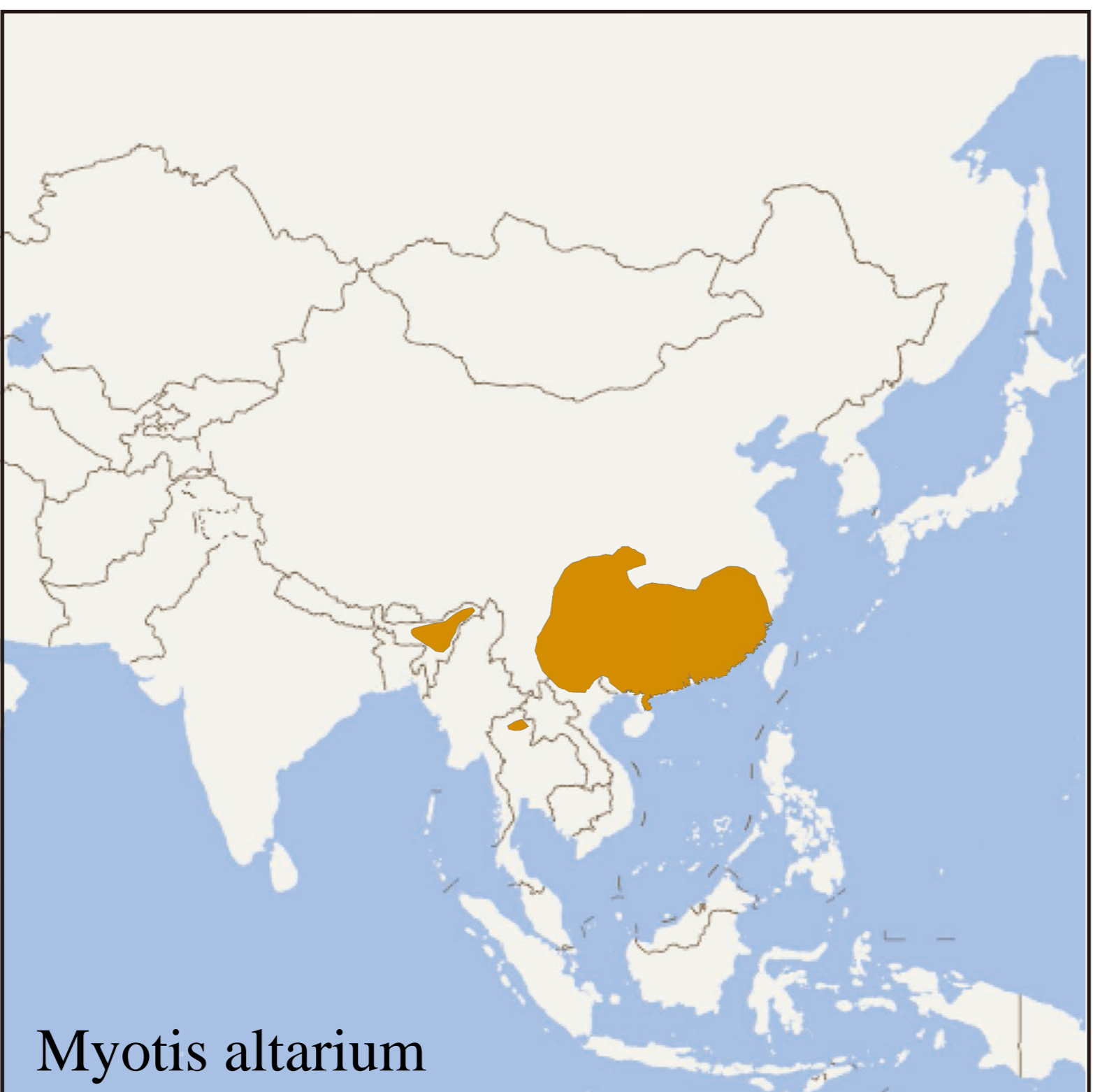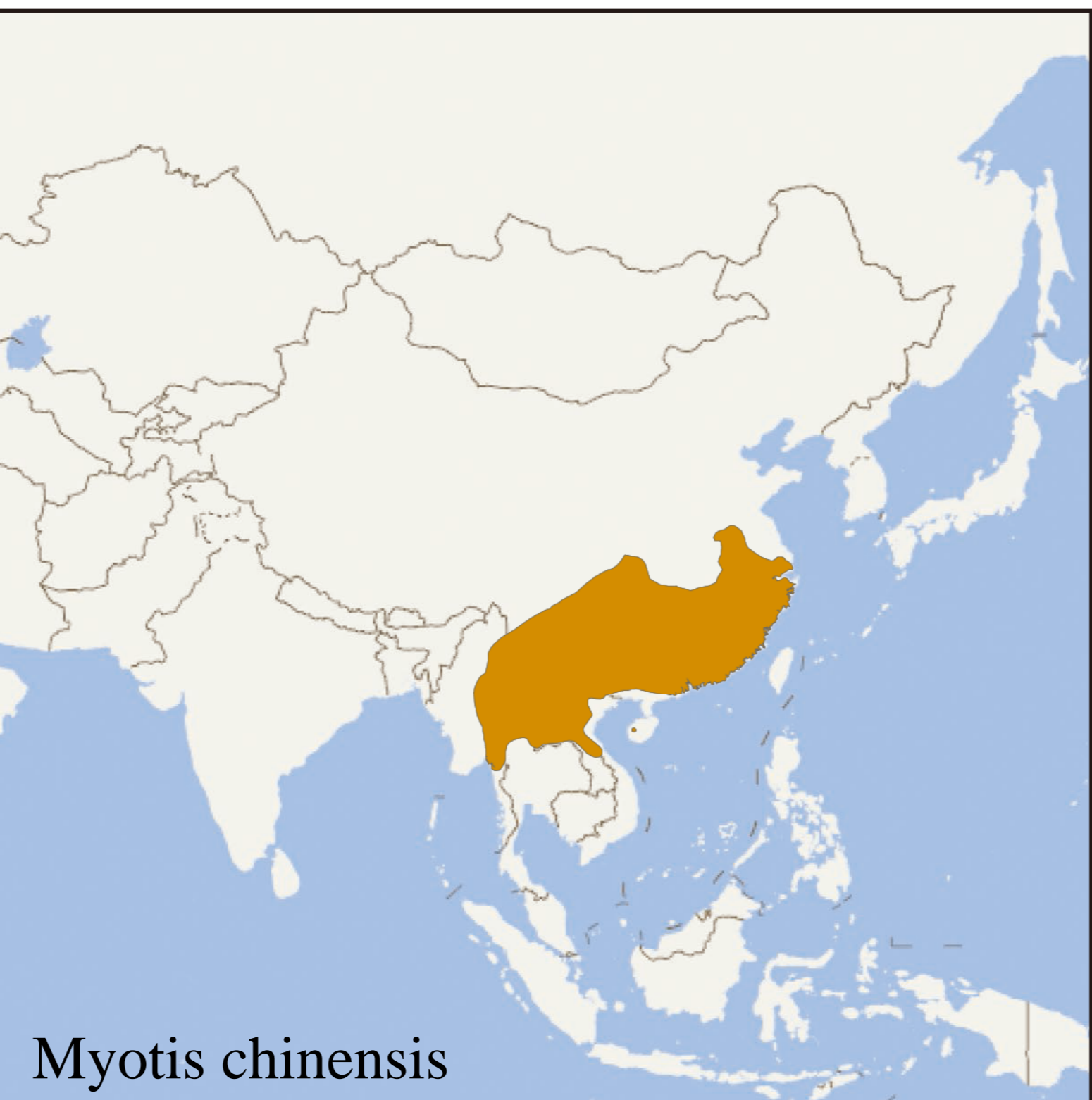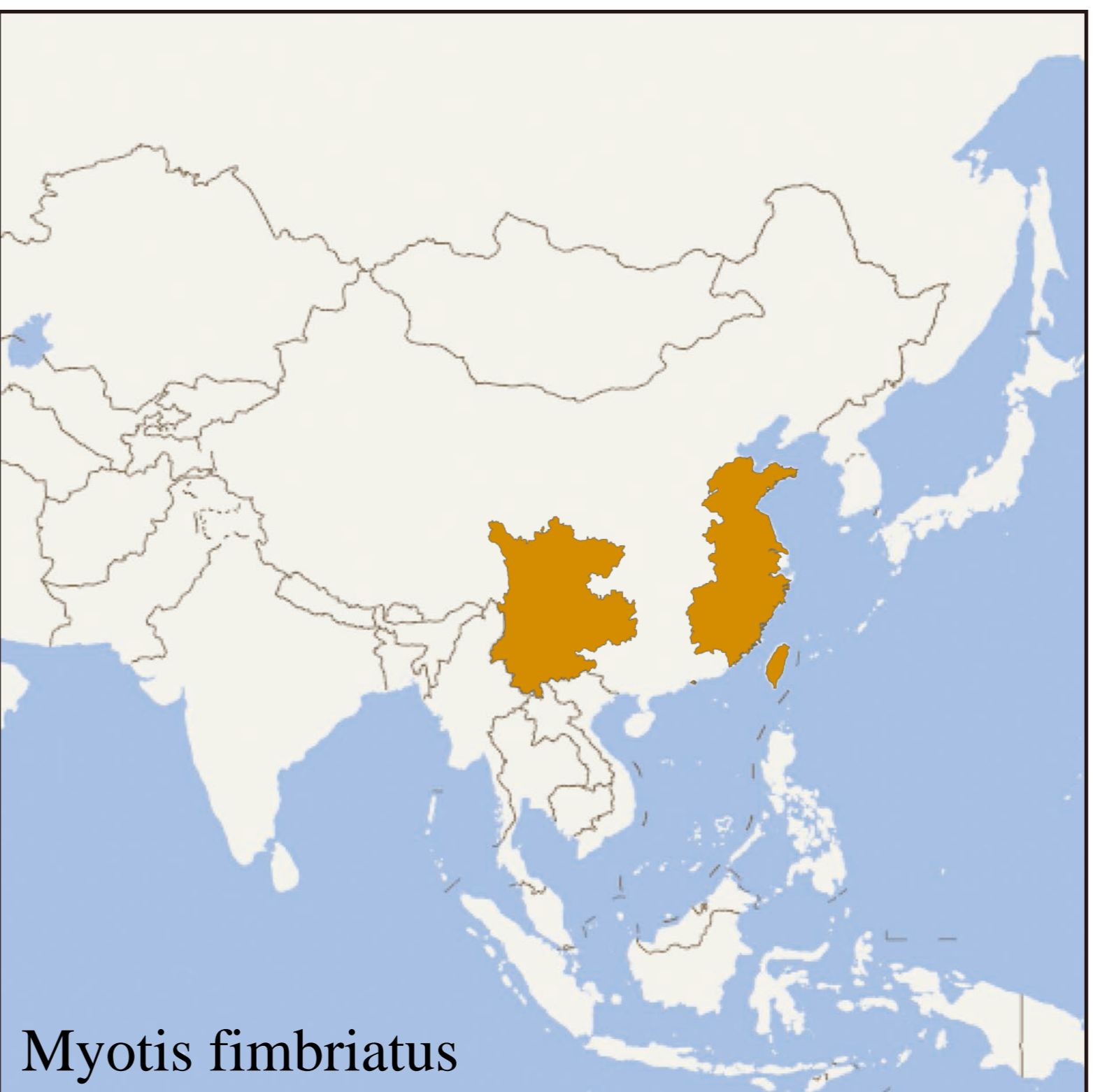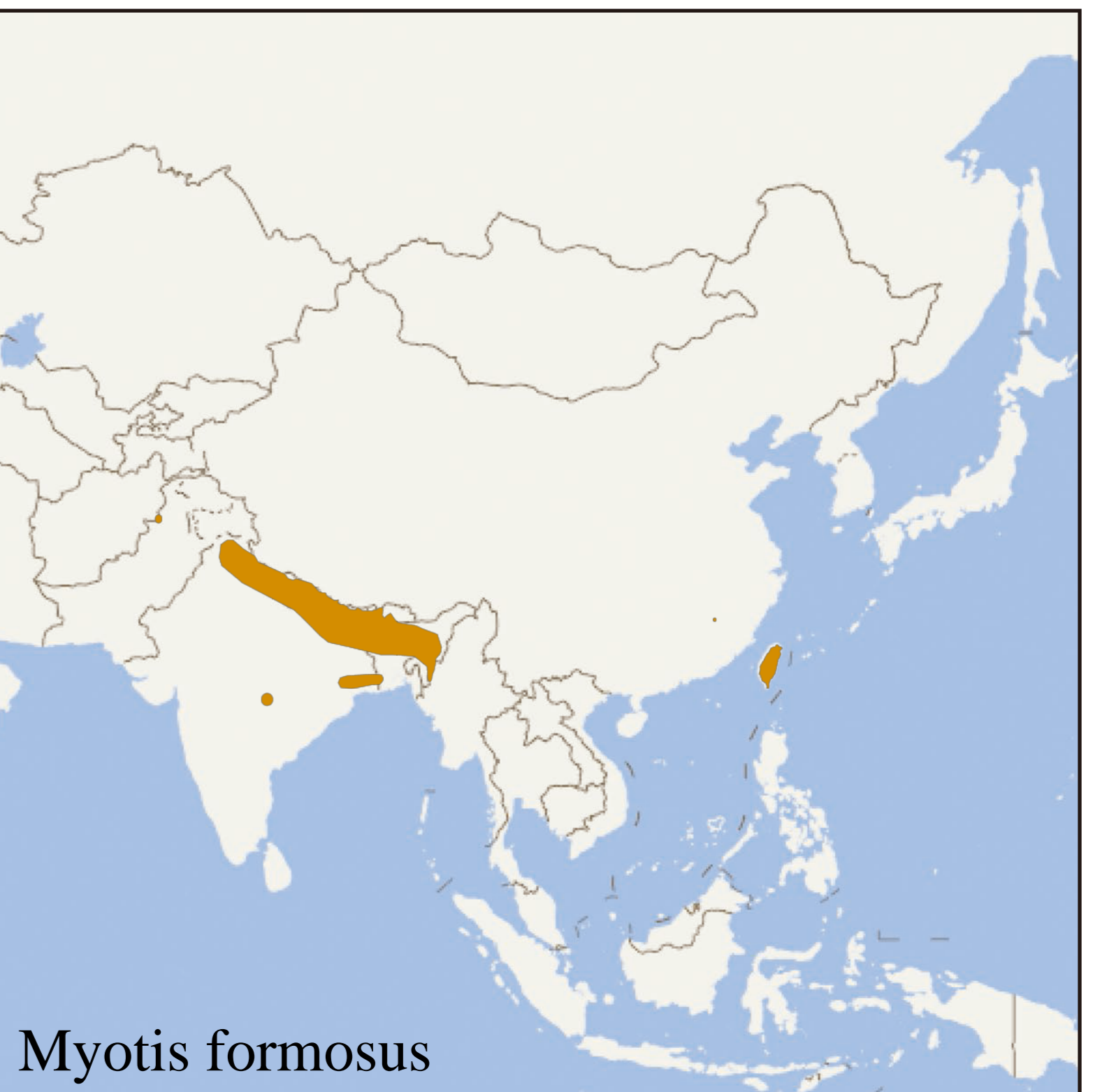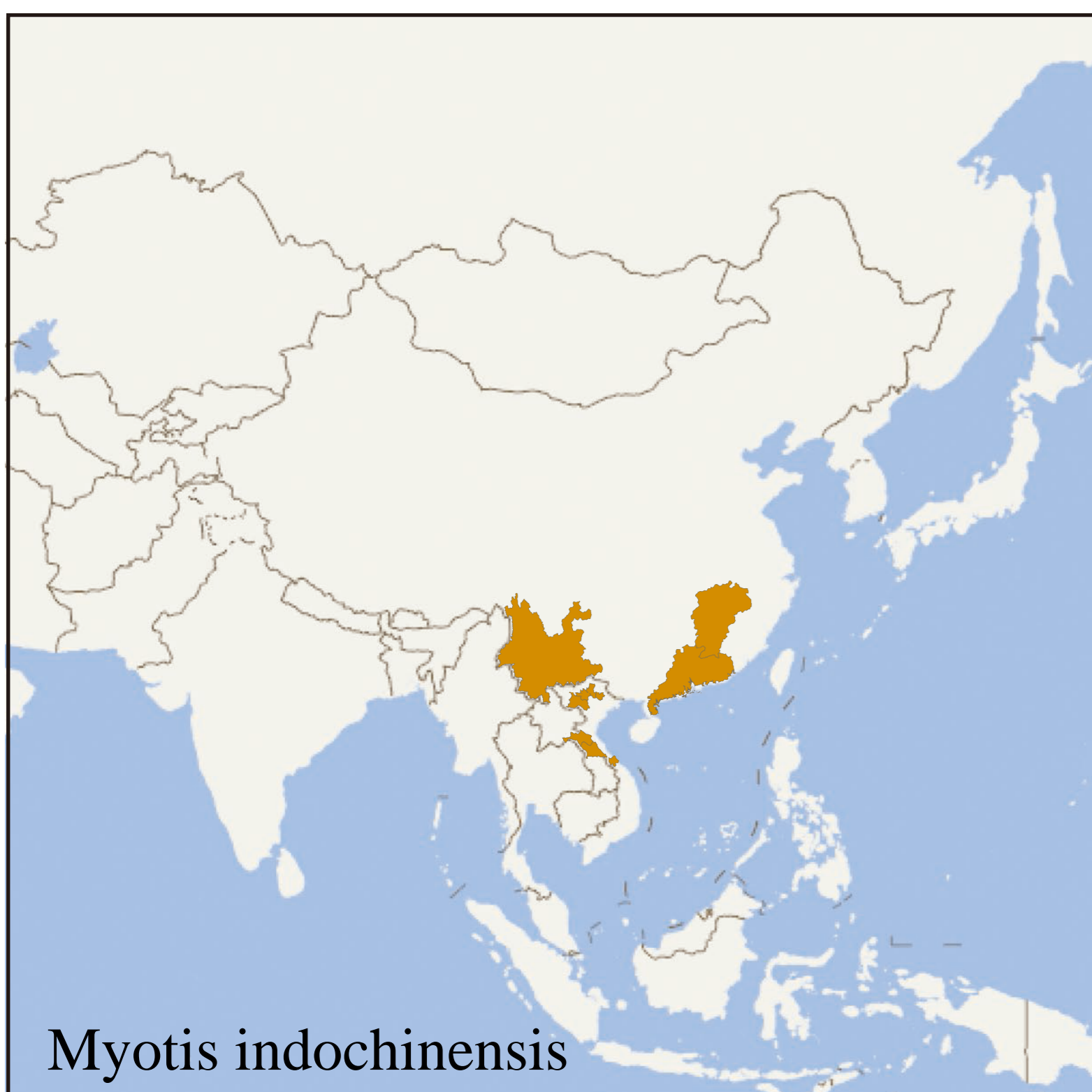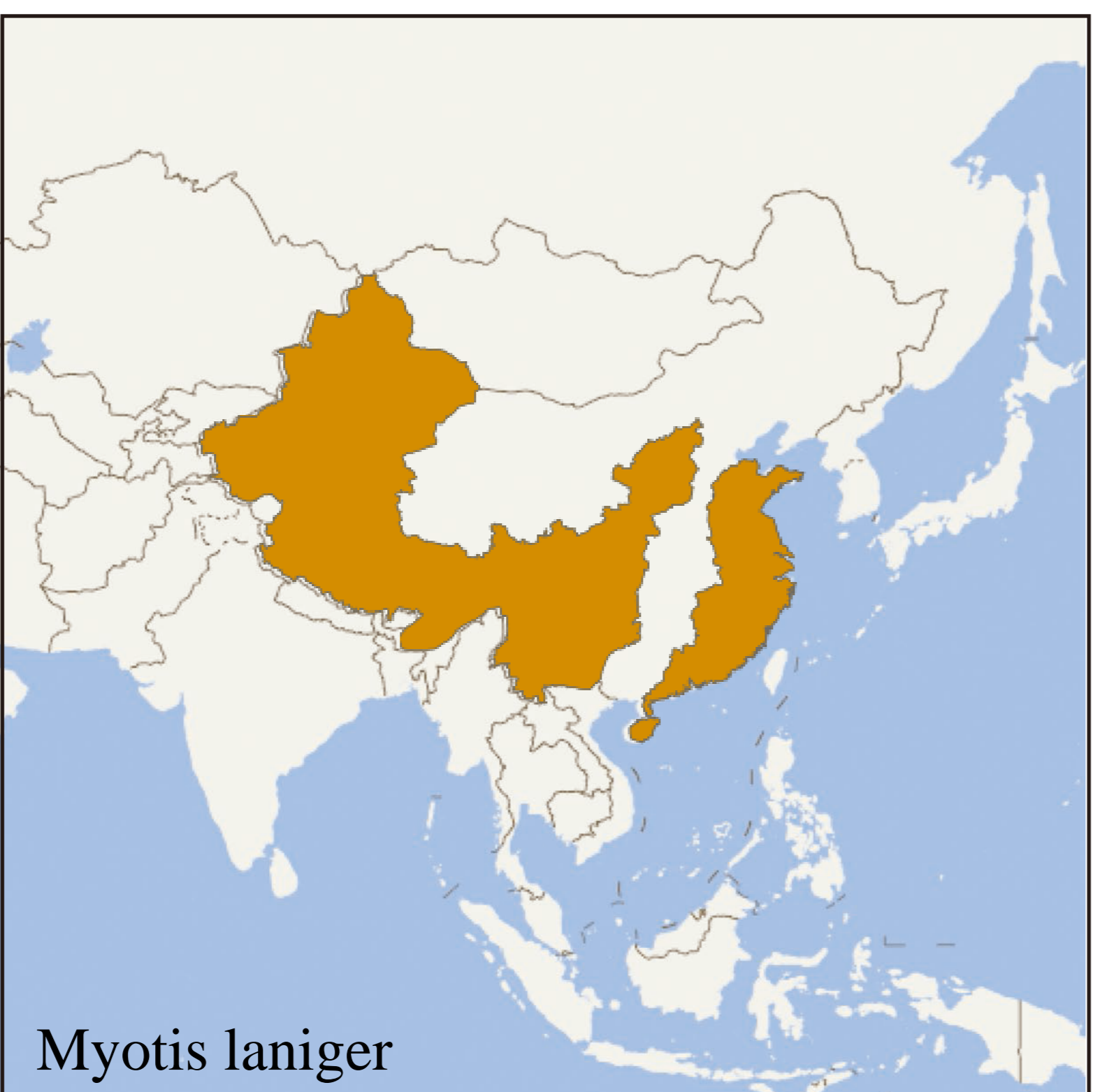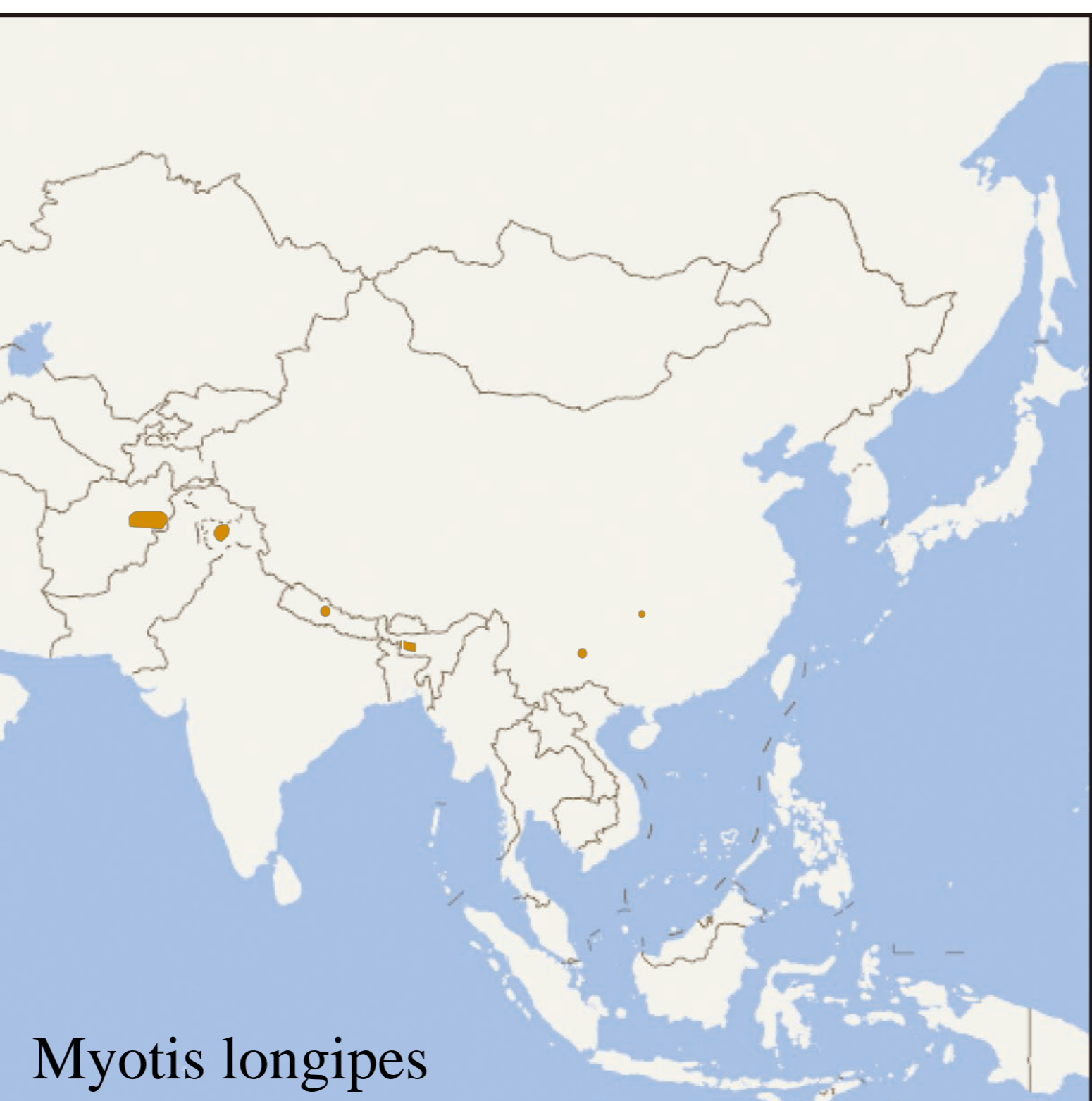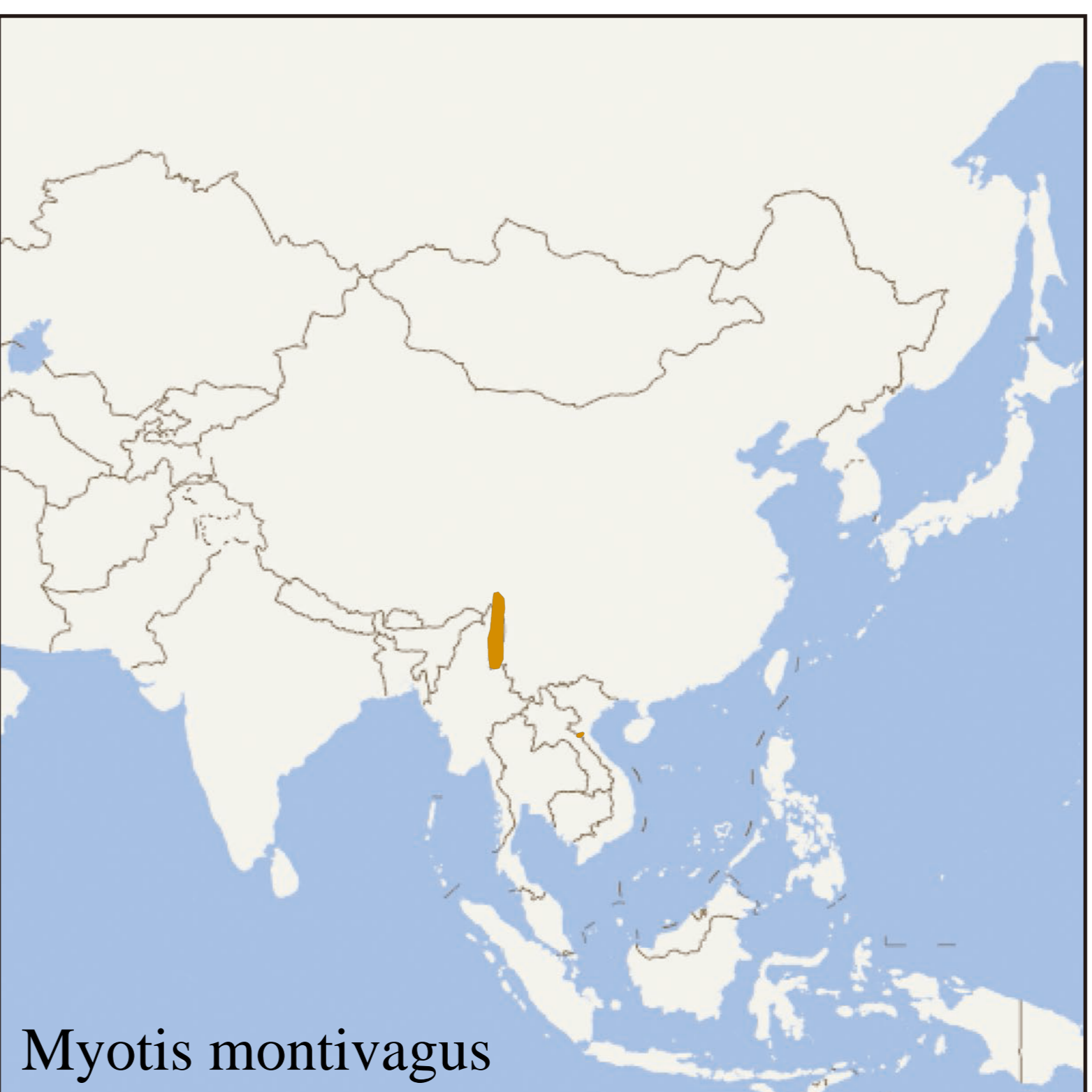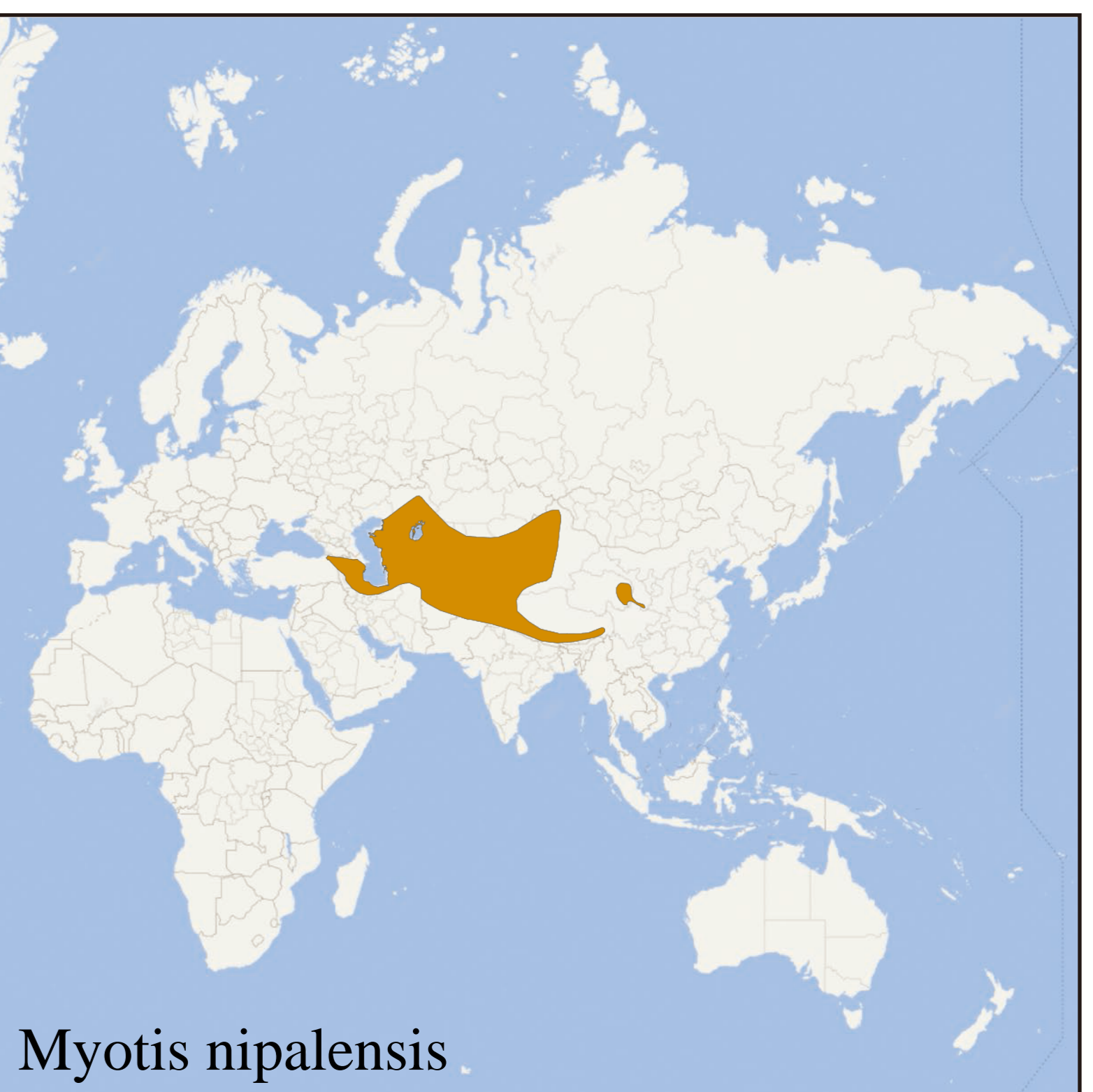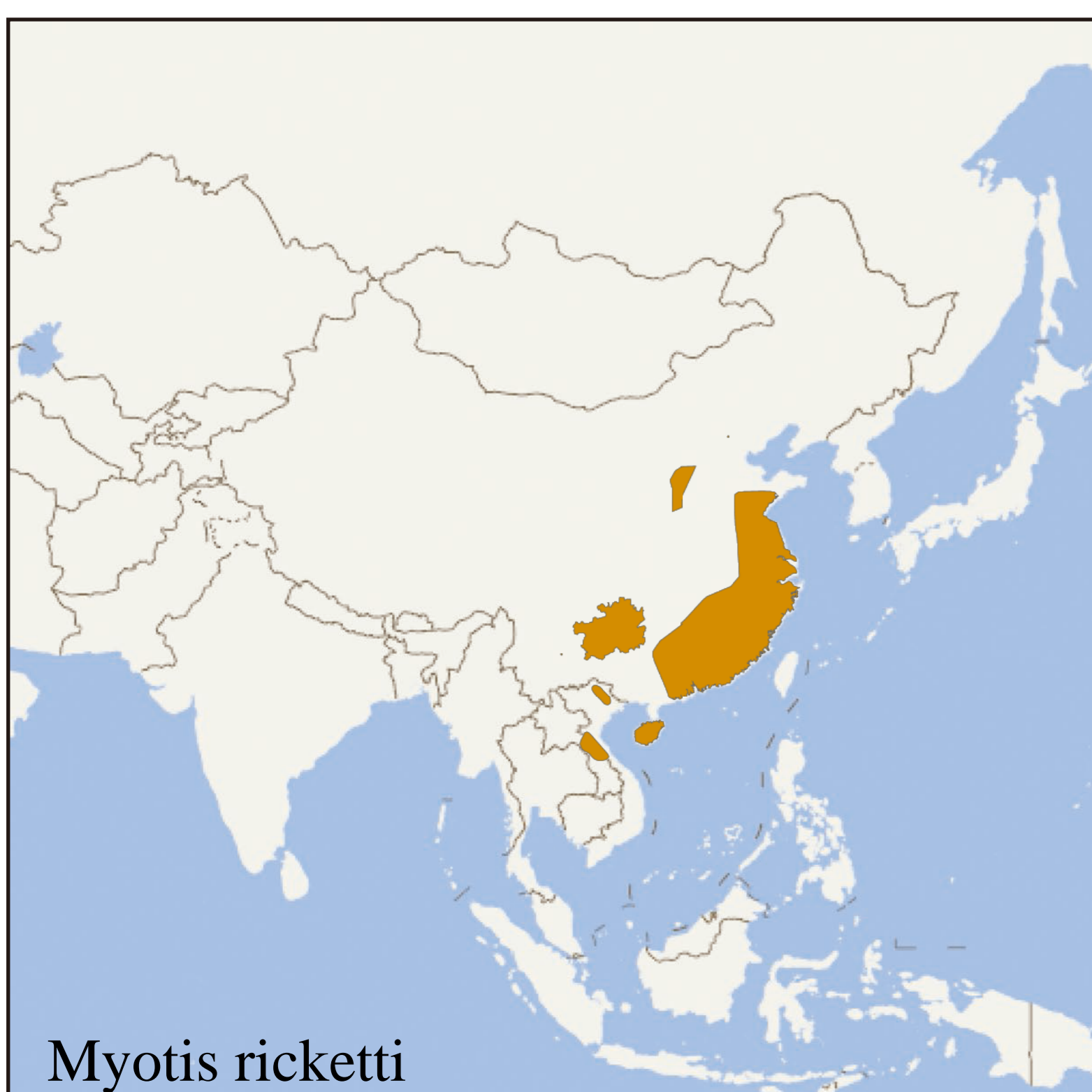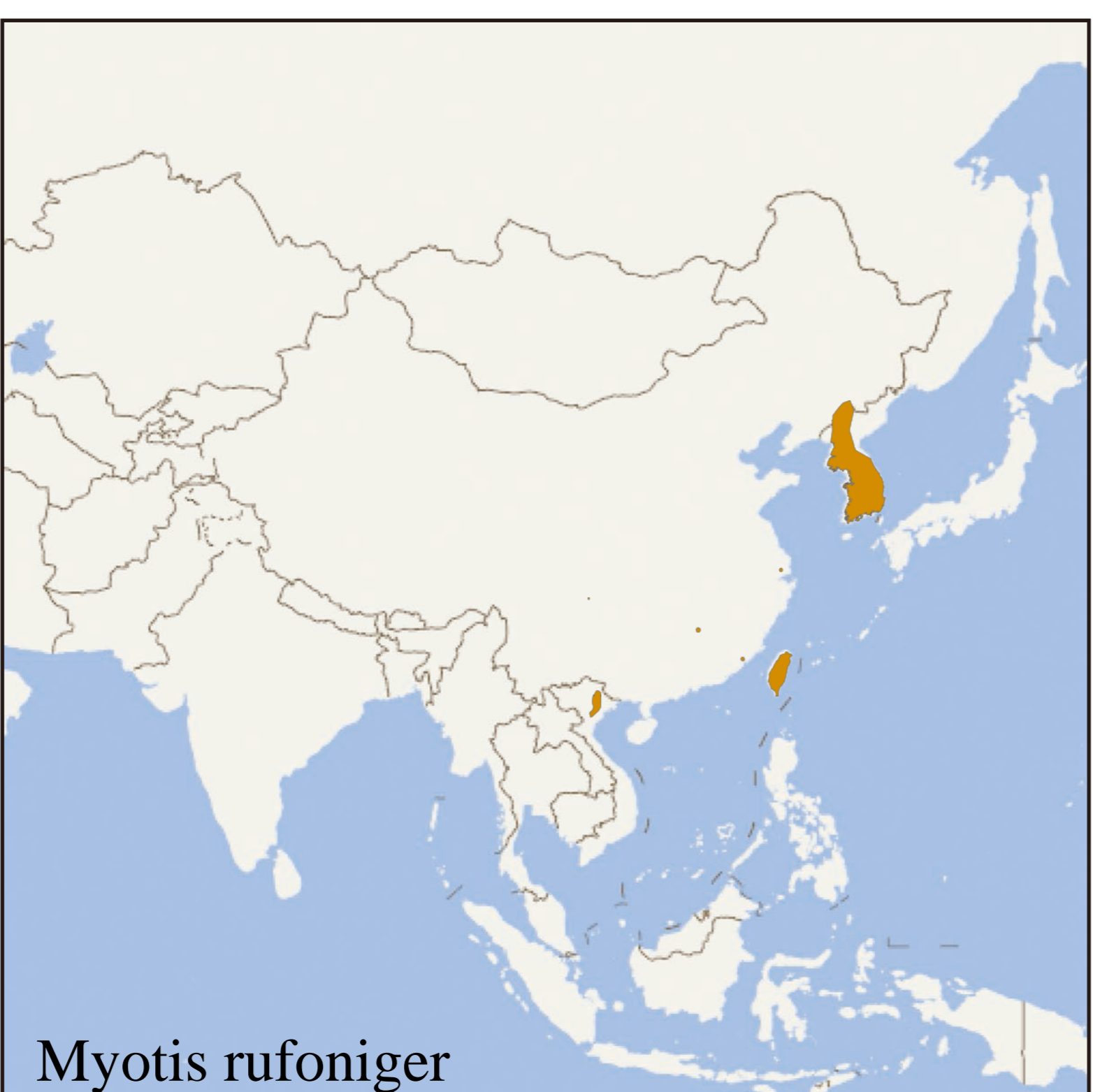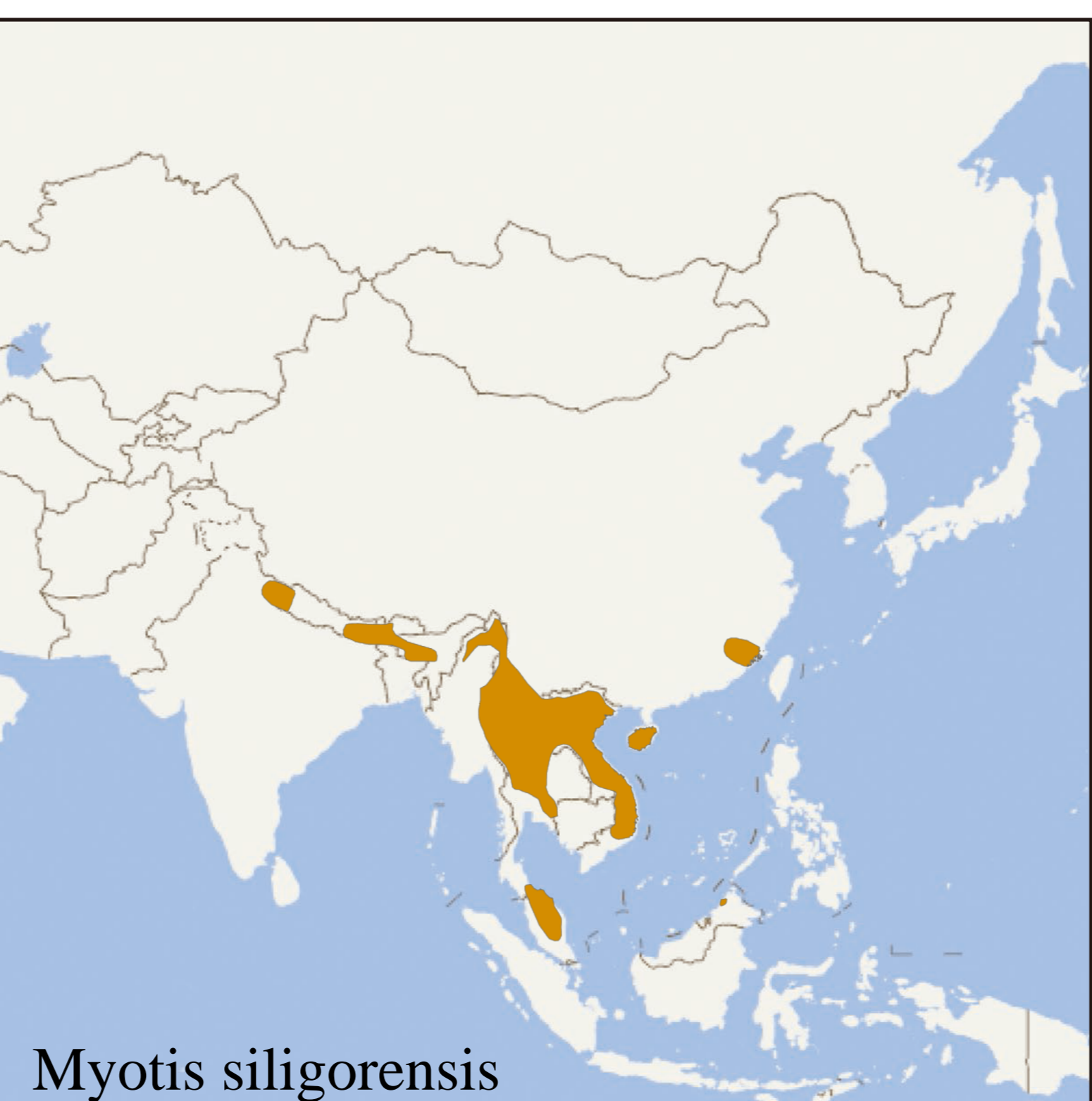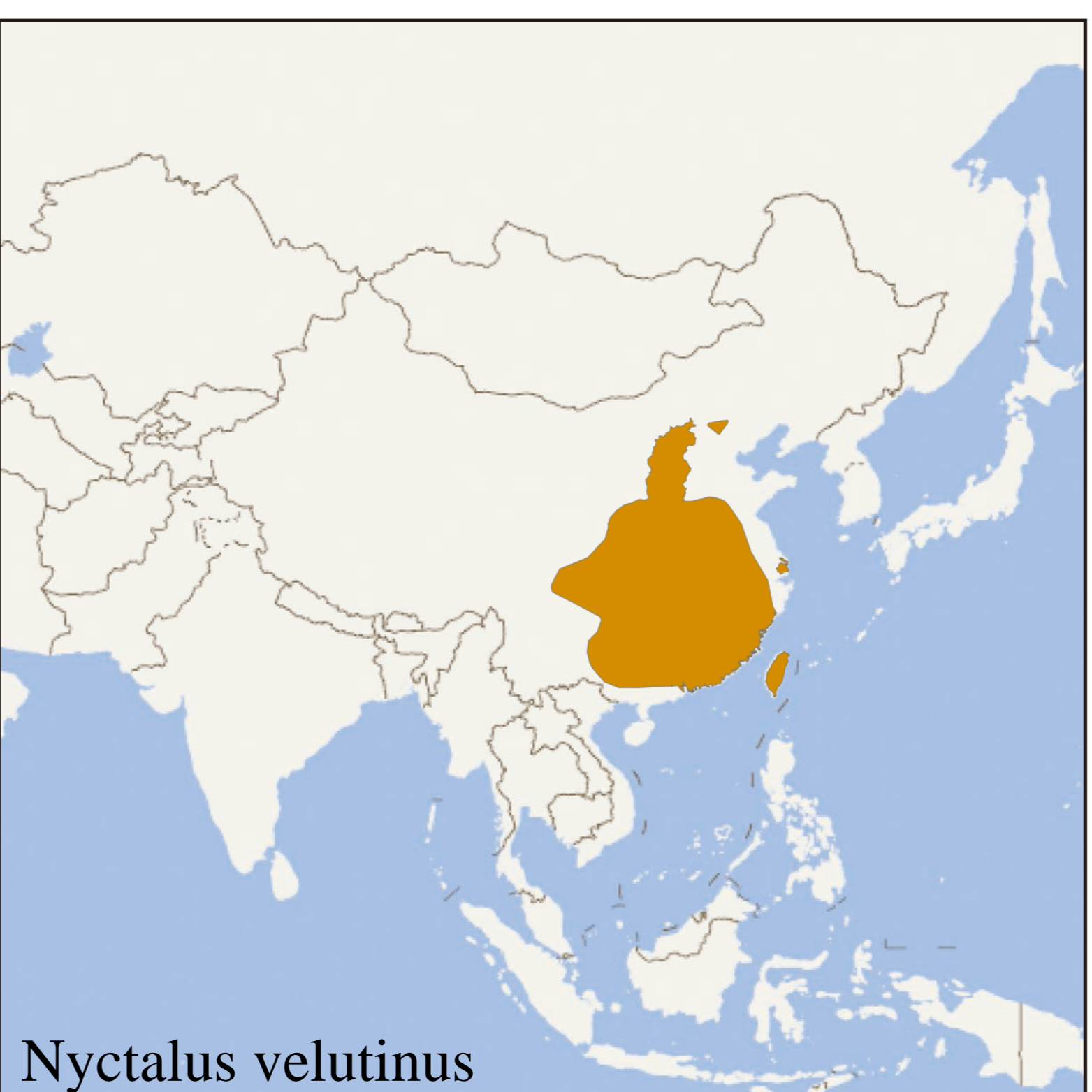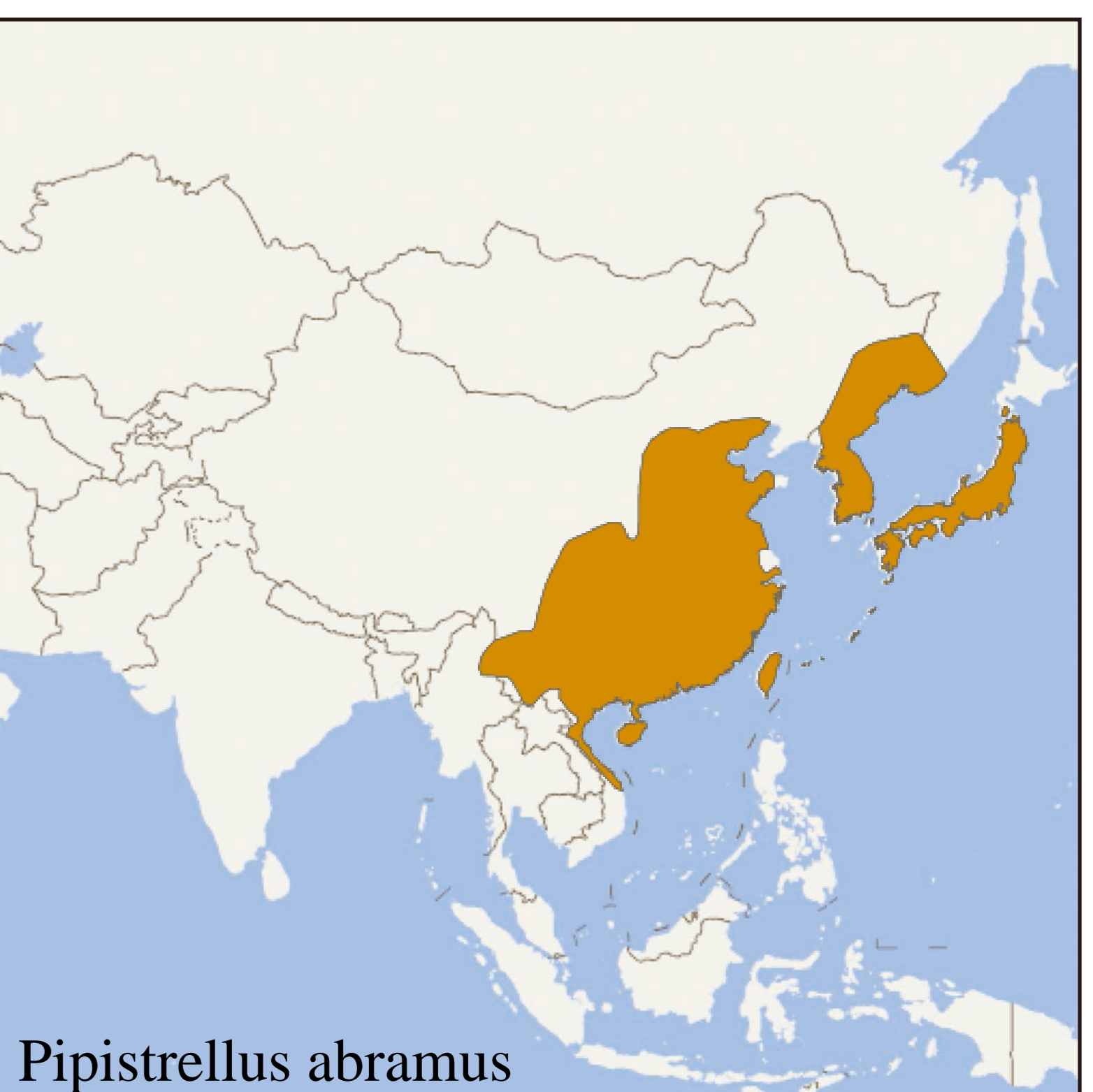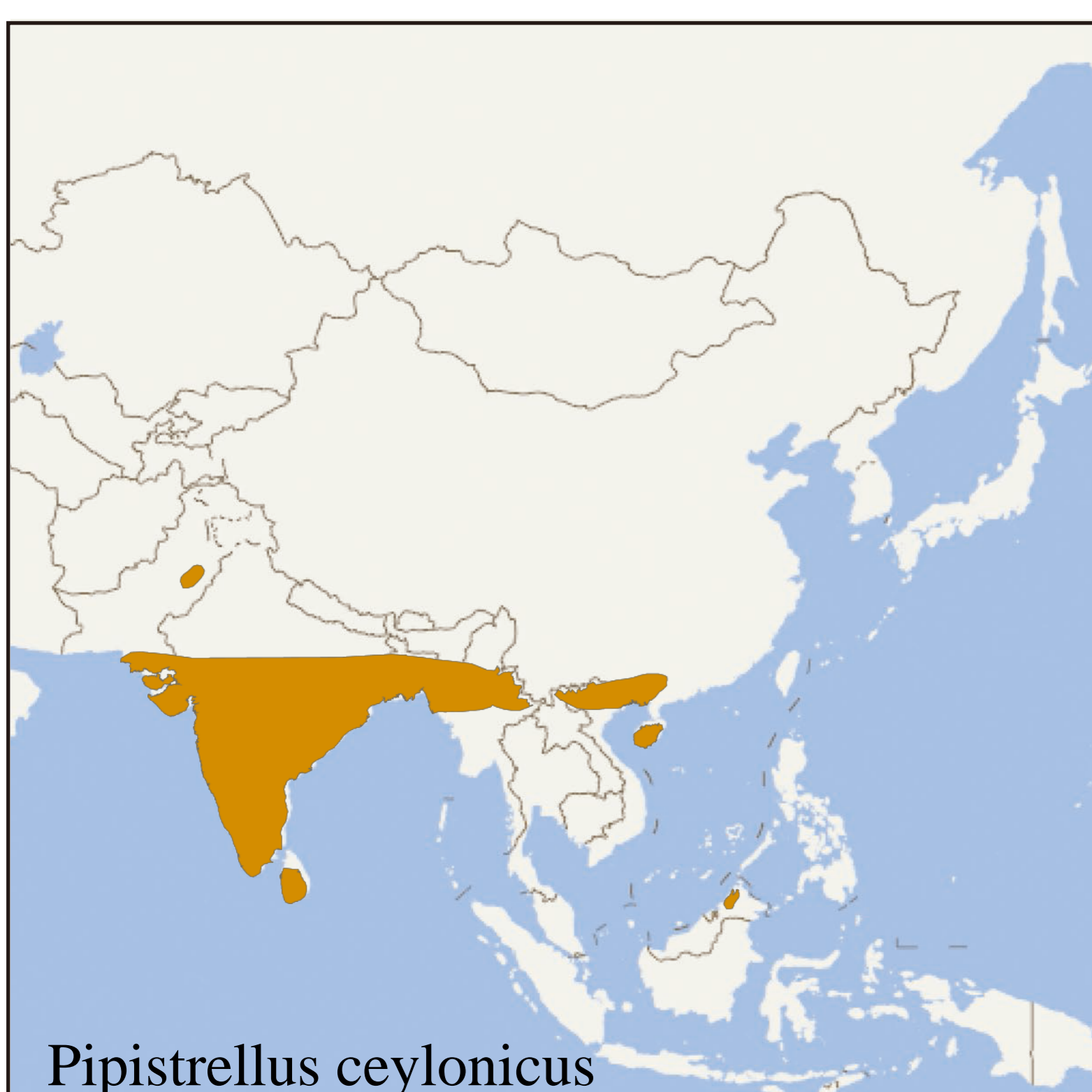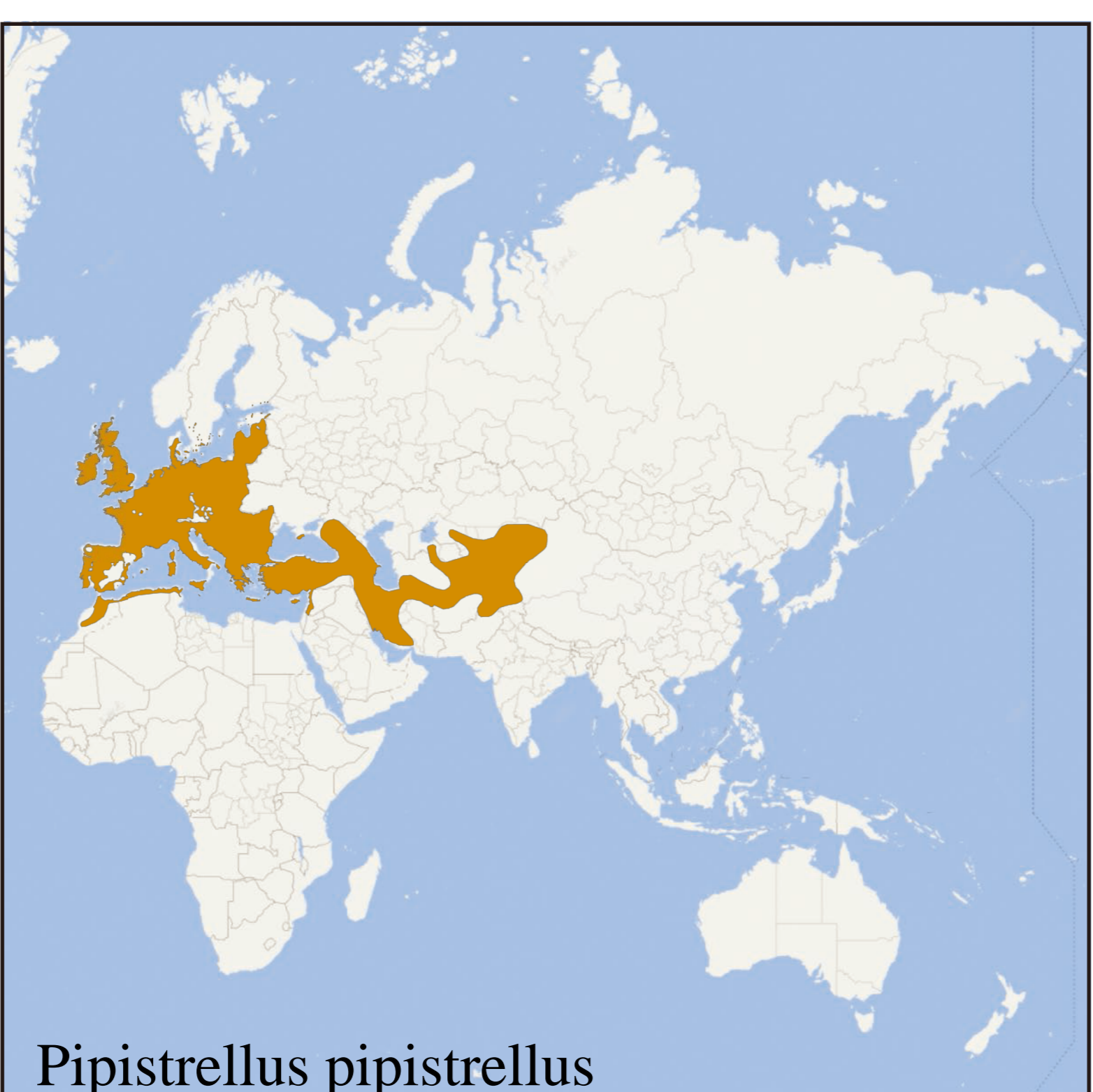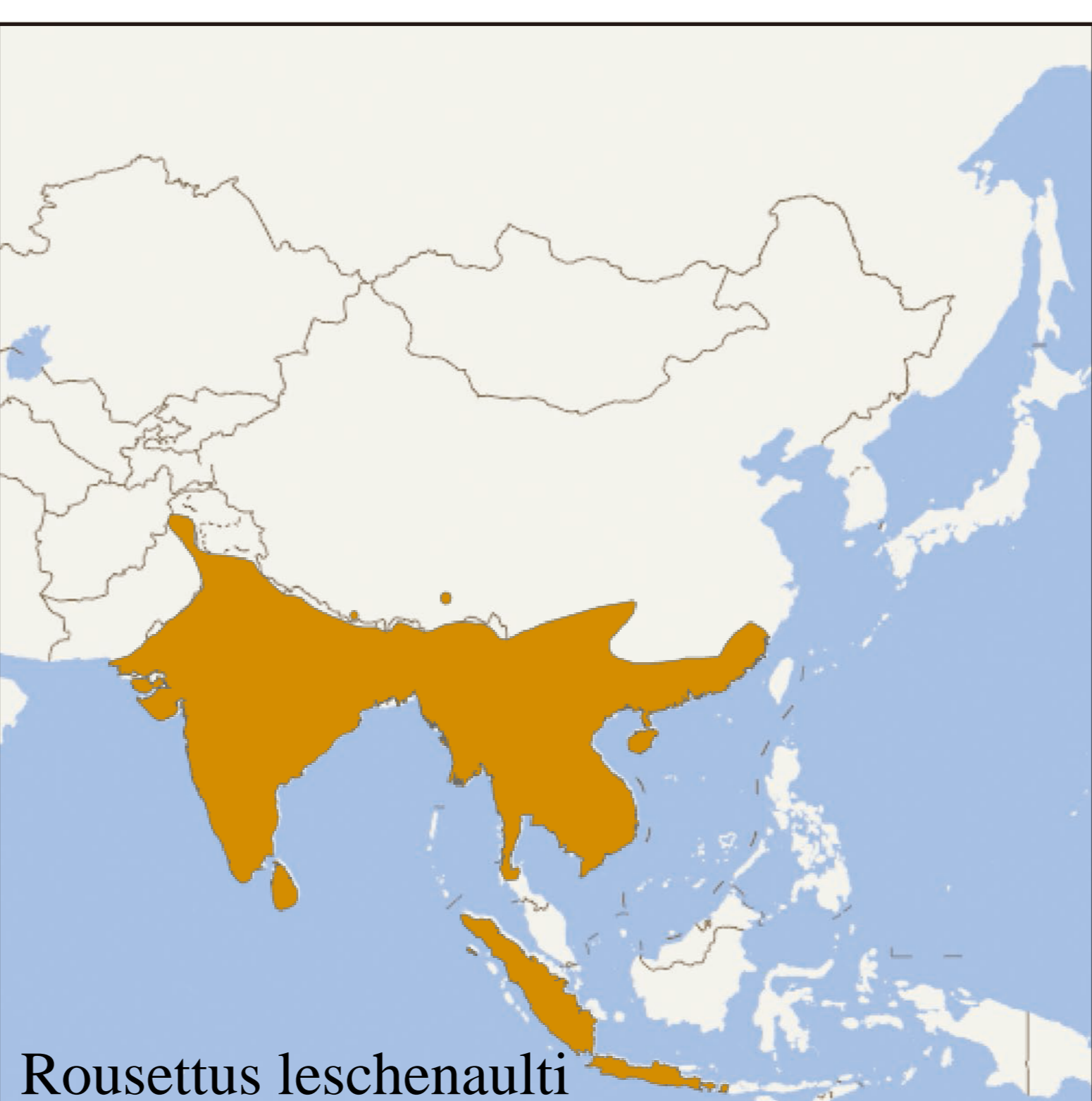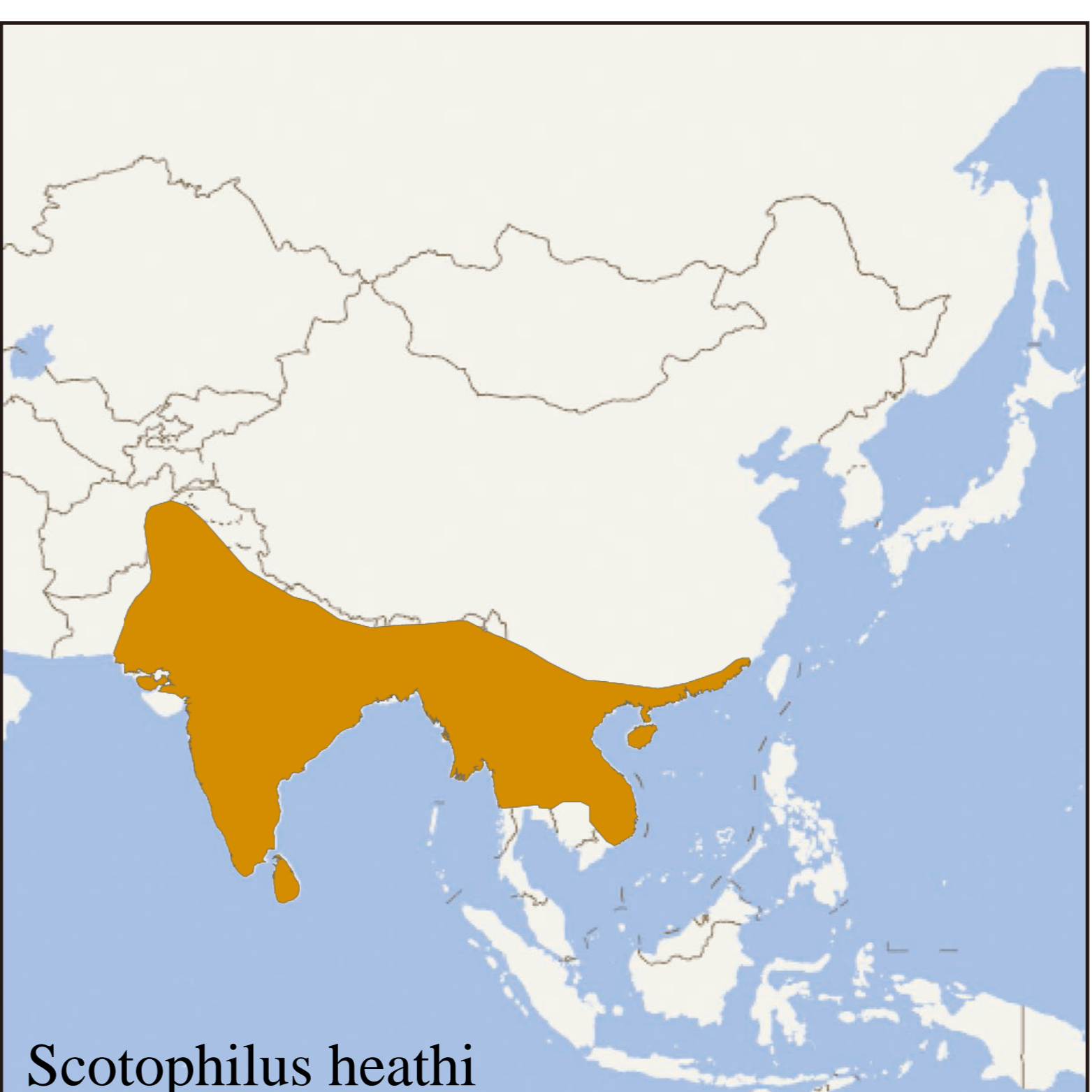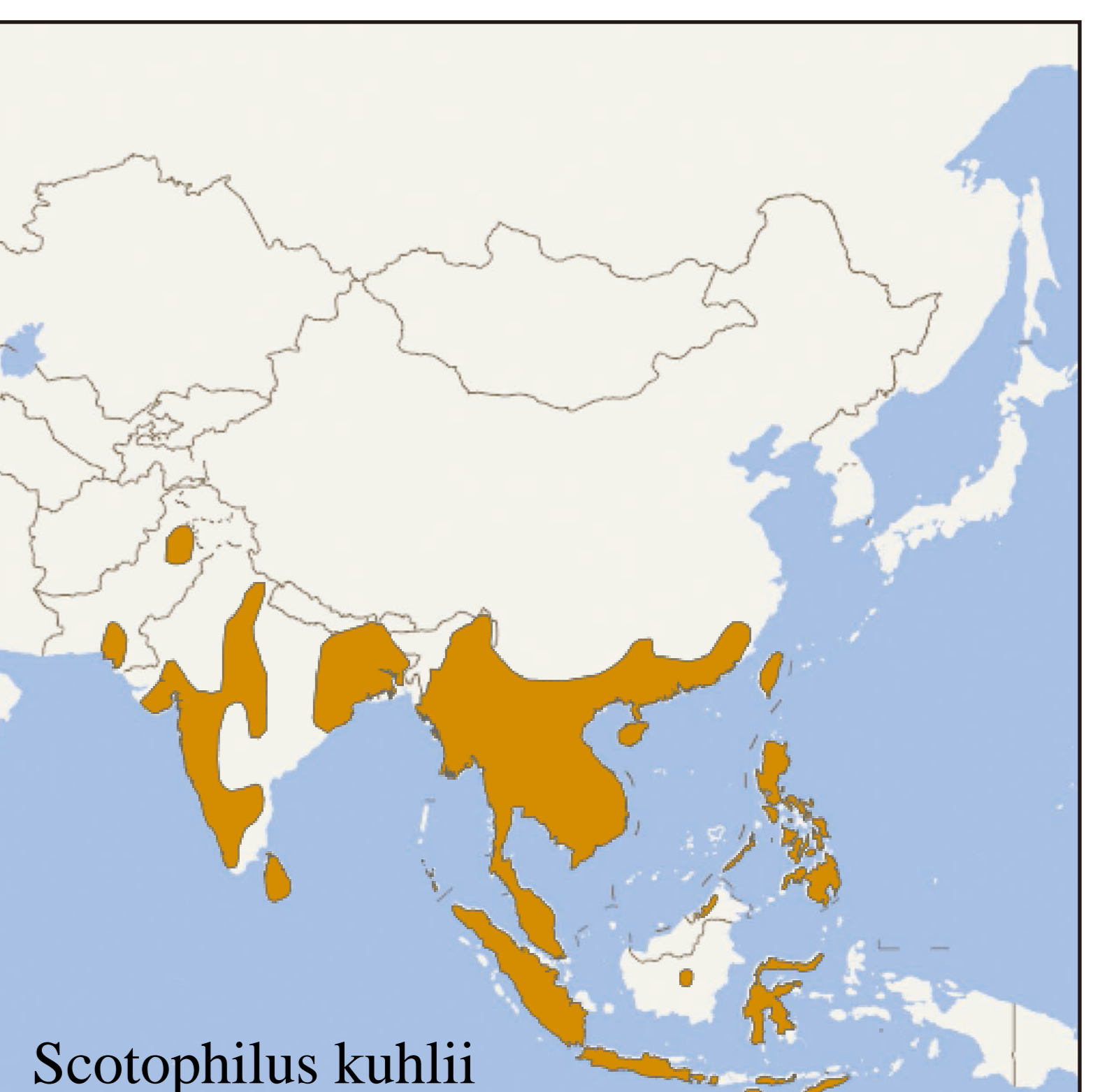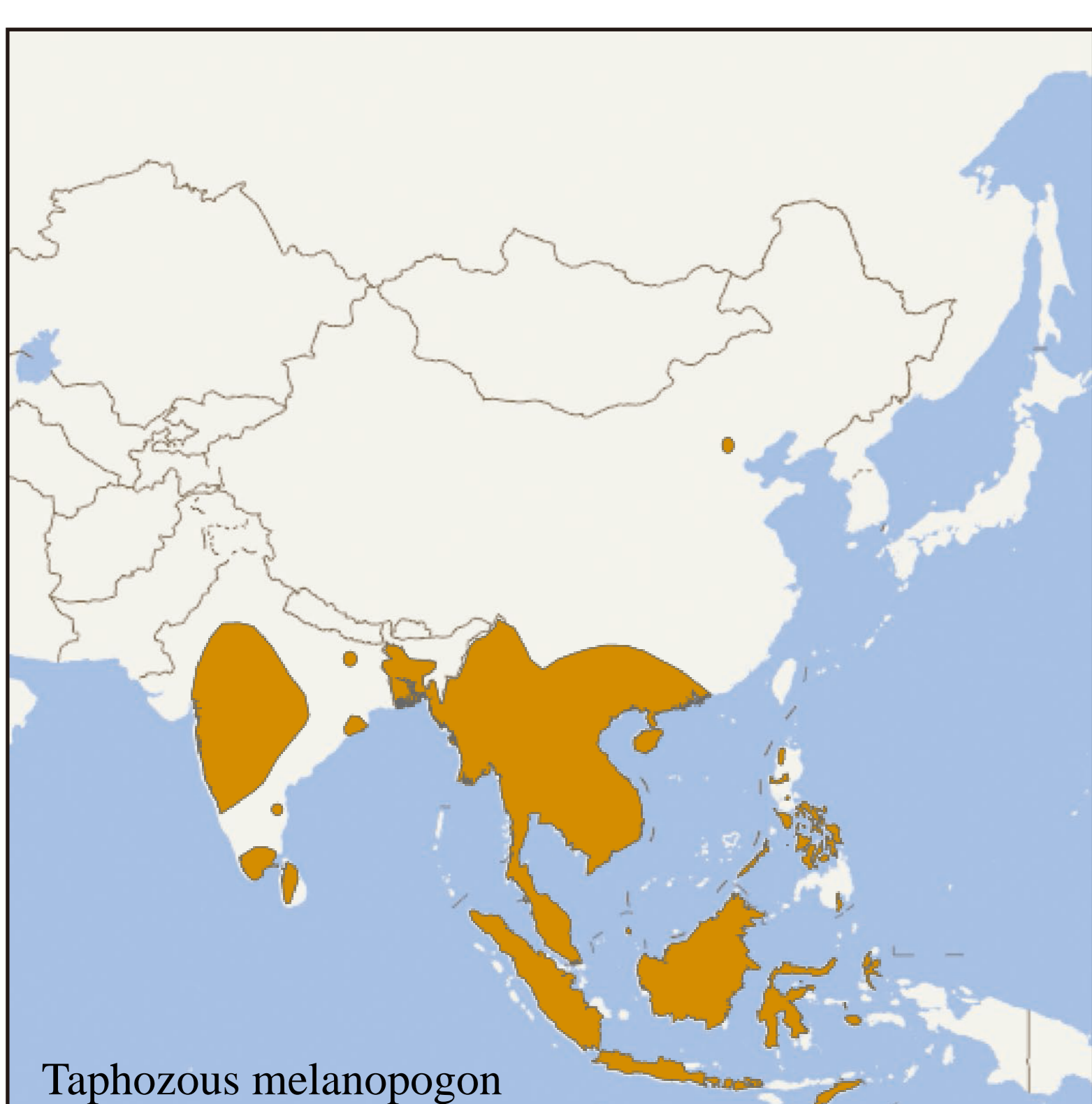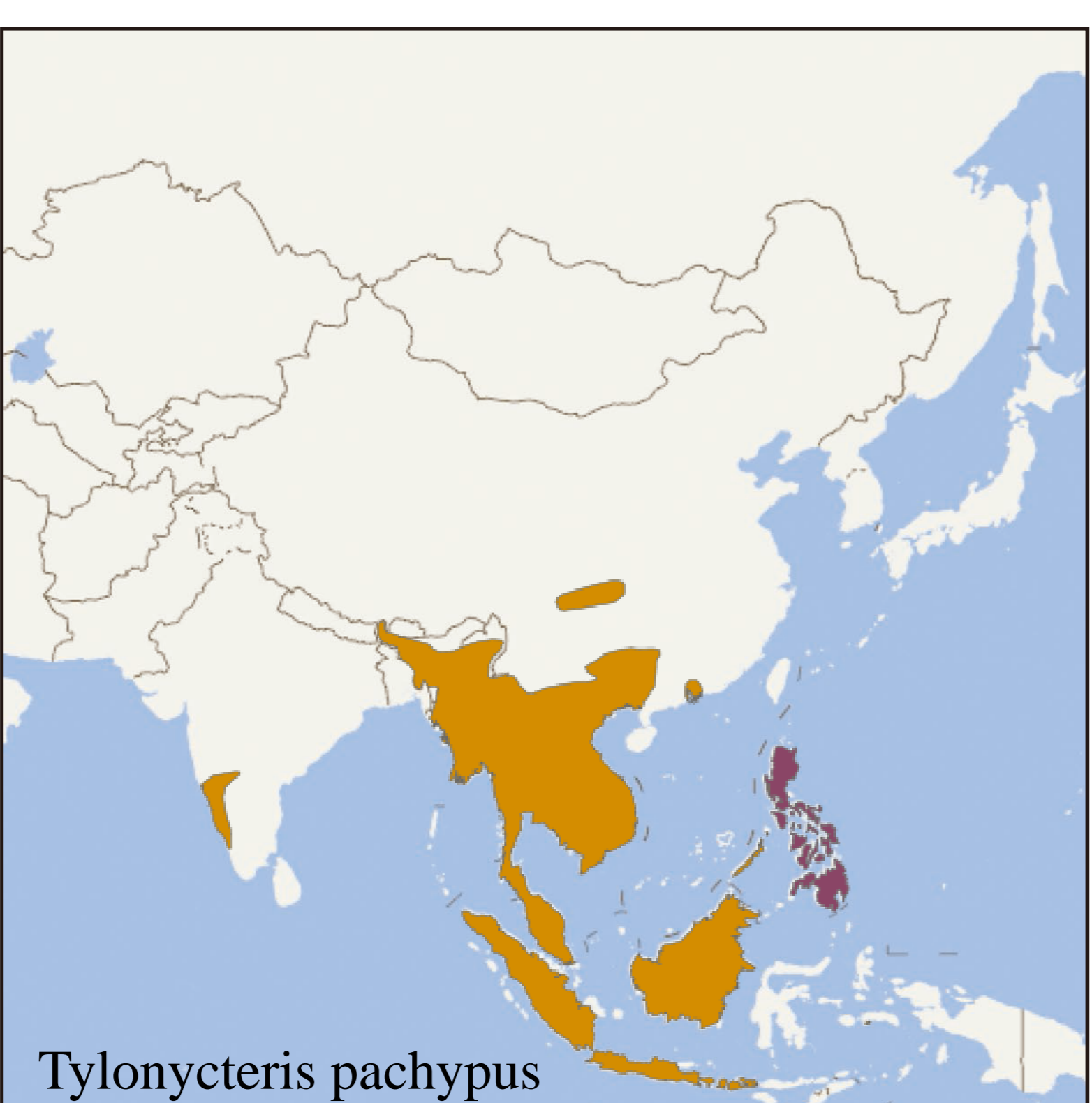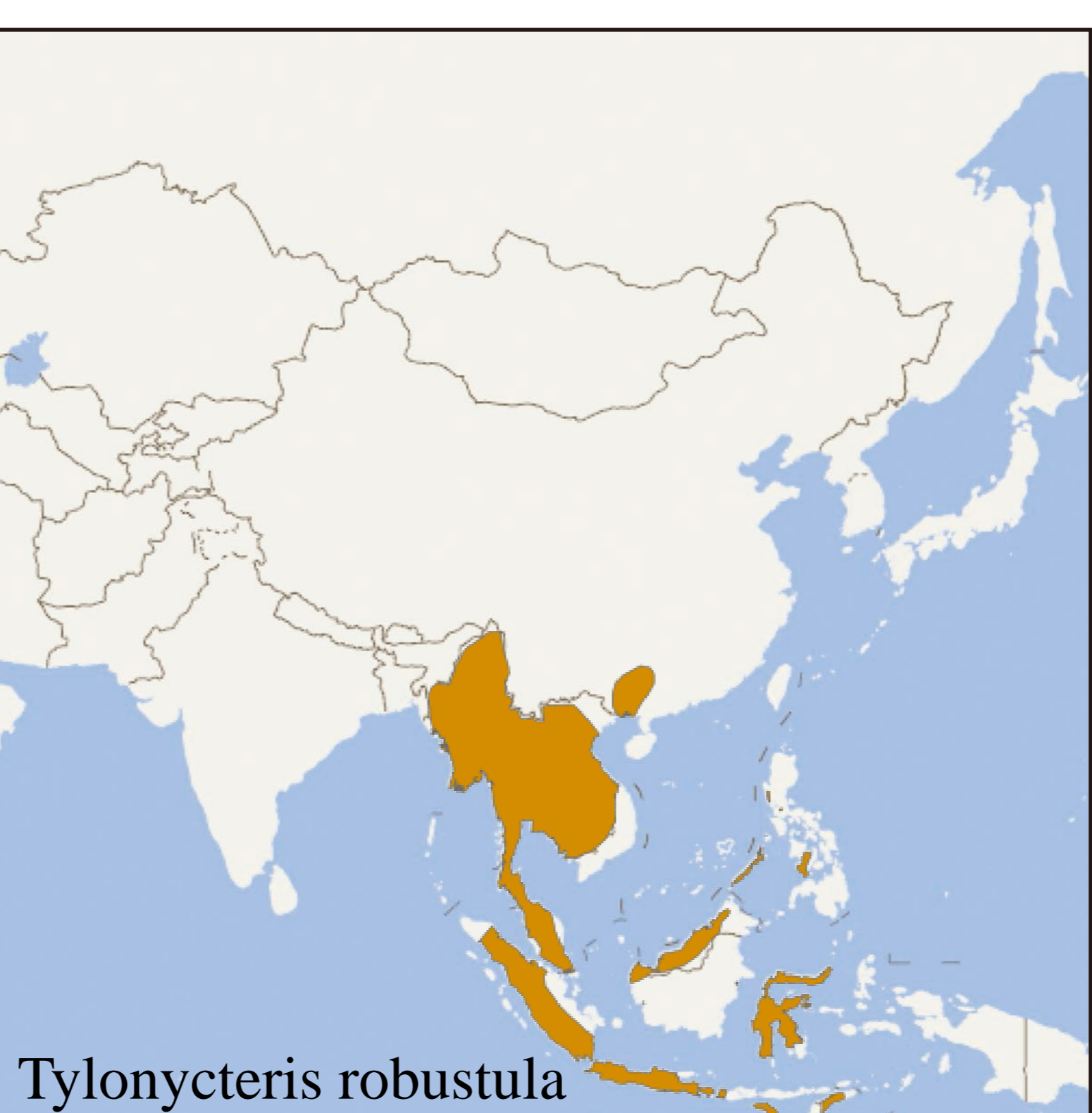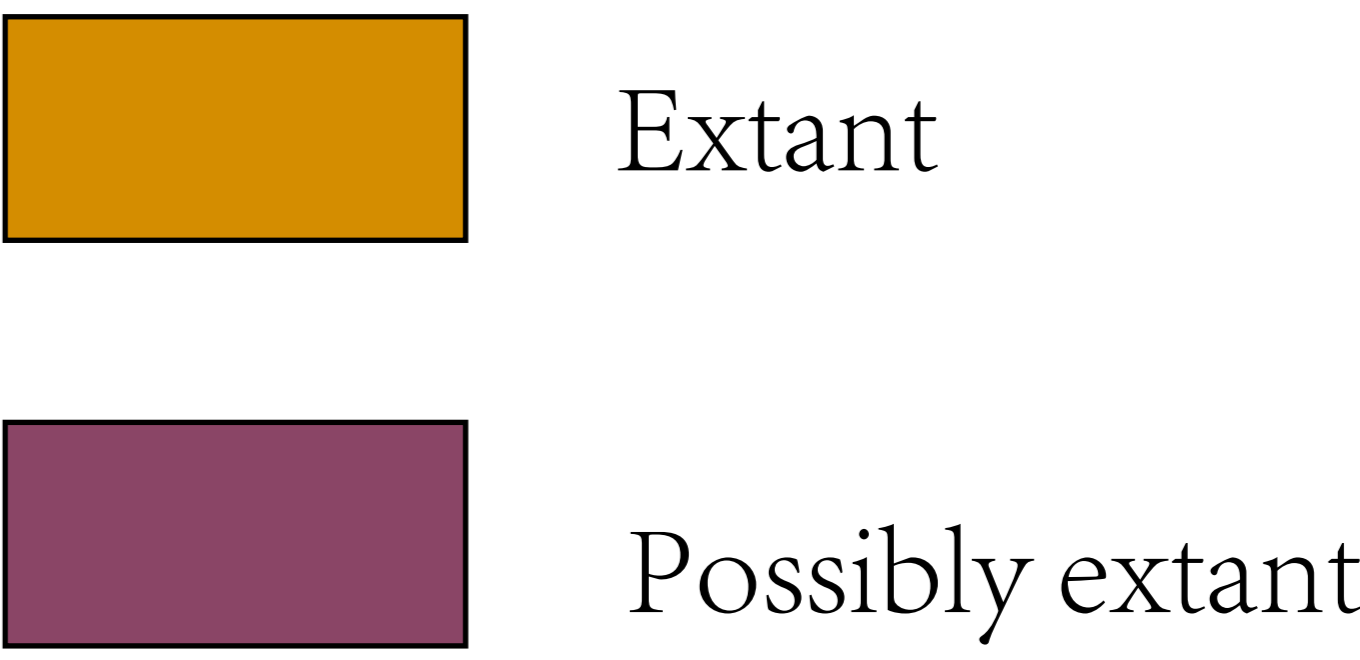

Supplement: nwac213_Supplemental_Files [file nwac213_supplemental_files.zip › Supplementary_Fig._1.pdf]

(A)

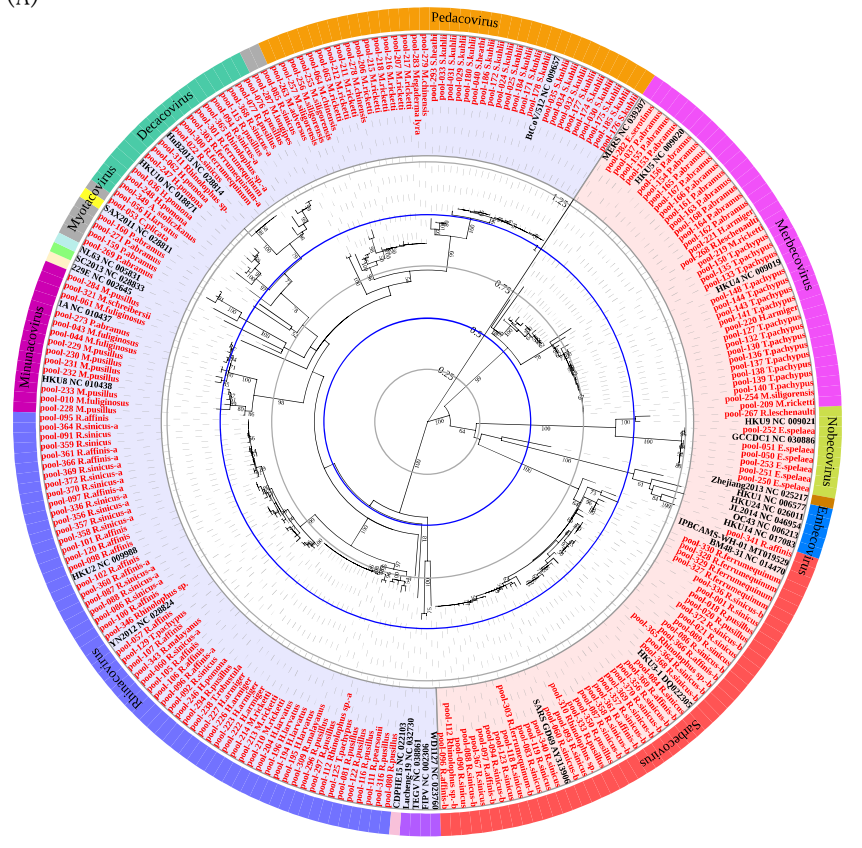

(C)

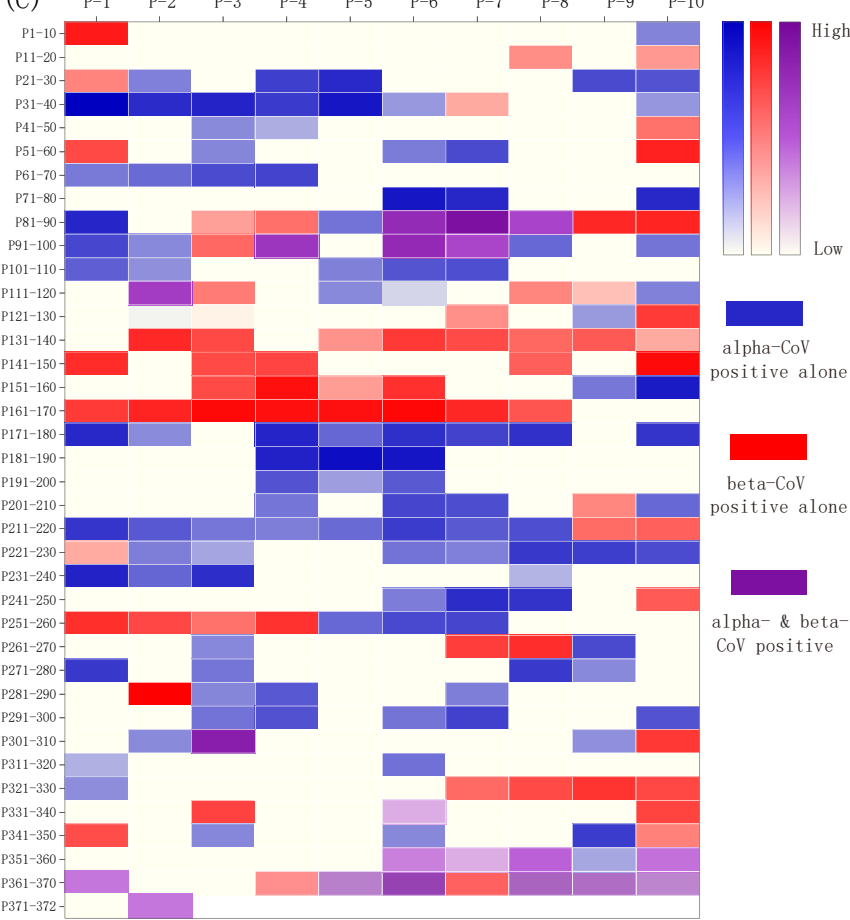

(B)

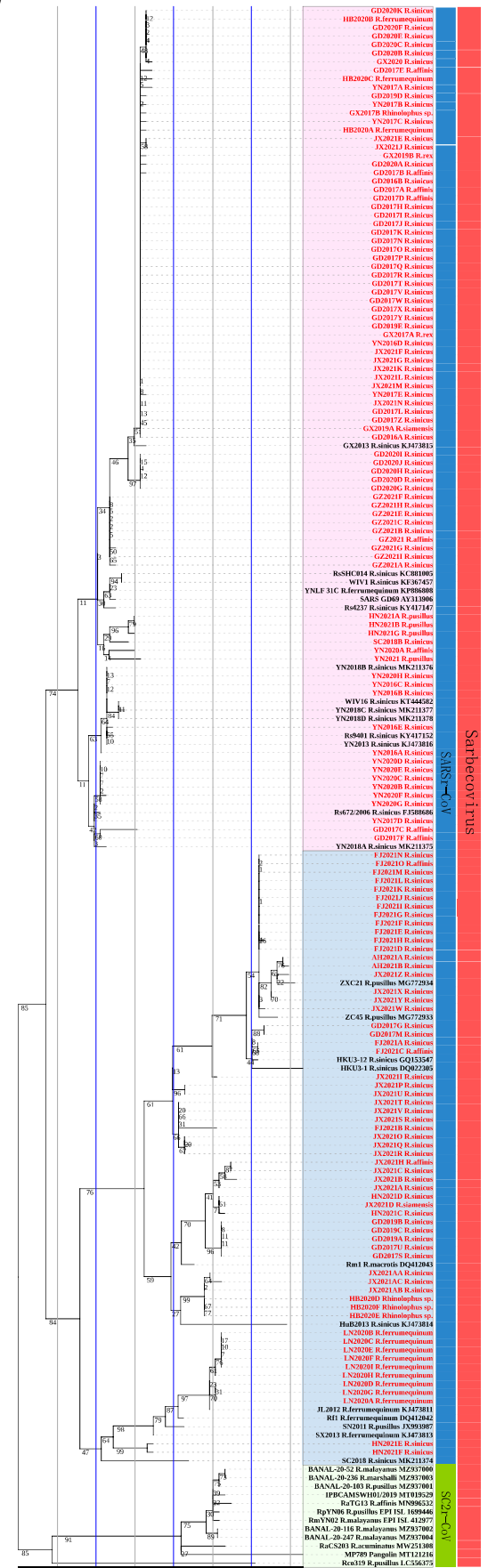

Supplement: nwac213_Supplemental_Files [file nwac213_supplemental_files.zip › Supplementary_Fig._2.pdf]
